# Structural, optical, electrical conductivity, and thermal properties of some mononuclear and mixed metal complexes of diethyldithiocarbamate

**DOI:** 10.1038/s41598-026-51751-0

**Published:** 2026-05-19

**Authors:** Rania Emara, Mamdouh S. Masoud, Sayed Abboudy, Ahmed M. Ramadan

**Affiliations:** 1 Chemistry Department, Faculty of Science, Alexandria University, Alexandria, Egypt; 2 Physics Department, Faculty of Science, Alexandria University, Alexandria, Egypt

**Keywords:** Chalcogenide metal complexes, Porous structure, Sellmeier model, Imaginary electric modulus, Conduction models, Debye model, Chemistry, Materials science, Nanoscience and technology, Physics

## Abstract

This study aims to synthesize and characterize a series of mononuclear and mixed metal diethyldithiocarbamate complexes containing Ag(I), Cu(II), Mn(II), and selenium ions, and to explore their structural, optical, electrical, and thermal properties. These complexes are of considerable interest because metal dithiocarbamates can serve as versatile single-source precursors for the fabrication of semiconductor materials and other advanced functional systems. The synthesized complexes were characterized using XRD, elemental analysis (SEM–EDX), spectroscopy techniques (IR, NMR, UV–Vis, ESR, and fluorescence), magnetic measurements, density functional theory (DFT), and thermal analyses (TGA, DTA, DSC). Structural investigations confirmed the ability of diethyldithiocarbamate ligands to form multinuclear coordination assemblies with the investigated metal ions, producing sponge-like structures. The UV–visible spectra showed strong absorption at 240–435 nm and high transmission (84%-99%) around 300 nm, with an optical band gap between 1.95 and 4.15 eV. The oscillator and the dispersion energies of linear refractive index (*n*) were evaluated using the Wemple Di-Domenico single oscillator model. The emission spectra exhibited three fluorescence peaks in the range 427–531 nm. The dielectric characteristics and alternating current conductivity (σ_ω_) were measured at temperatures ranging from 298 to 400 K and frequencies between 120 Hz and 100 kHz. The electrical measurements of the samples revealed semiconducting behavior with *σ*_*ω*_ values of 10^–7^–10^–1^ S/m, and *E*_*a*_ of 0.035–2.71 eV. Various conduction mechanisms were observed with increasing temperature, as evidenced by changes in the dielectric parameters. Thermal analyses emphasized that complexes may be applied for the synthesis of nanoscale metal sulfides with semiconductor properties.

**Supplementary Information:**

The online version contains supplementary material available at 10.1038/s41598-026-51751-0.

## Introduction

Dithiocarbamates are an interesting class of functional groups with various applications in agriculture, medicine, synthetic organic, analytical, coordination chemistry, materials science, and separation processes^1^. They also act as significant dithiolate ligands owing to their distinctive magnetic and spectral characteristic, facile redox behavior, and ability to stabilize metal ions in normal oxidation states^2,3^. Diethyldithiocarbamate (Et_2_DTC) is capable of stabilizing both transition and non-transition metals across a wide range of oxidation states due to the existence of thioureide resonance forms and extensive electron delocalization between N and S atoms as well as with metal ions^4,5^. Et_2_DTC has also been applied in the treatment of heavy metal intoxication, as toxic metals can generate mutagenic and carcinogenic chemicals in the body even at extremely low concentrations^5,6^.

Chalcogenide (S, Se, and Te) lone-pair semiconducting materials are significant because of their potential use in topological insulators, phase-change memory, thermoelectric, and other fields. The presence of lone pairs reduces strain energy in the system, allowing chalcogenide atoms to form amorphous structures by combining with other elements^7^. Sulfur-based metal complexes act as precursors for the designing and fabrication of semiconductors materials, thin films, alloys, and polymer composites. In addition, chalcogenides-containing precursors particularly those incorporating selenium and tellurium have a variety of uses in electrical, photoelectric, thermoelectric, and optoelectronic devices^8^. The structure stability and controlled thermal decomposition of metal complexes make them promising precursors for the synthesis of metal chalcogenides^9^. Reported metal chelates of dithiocarbamates have been successfully used as a single source precursor to synthesize binary metal sulfide semiconductors (such as ZnS and CdS)^10^, as well as ternary, quaternary, and multinary metal sulfides with potential uses in a variety of fields, such as solar cell applications, energy storage, imaging, and water purification^9,11^. In general, metal complexes exhibit intriguing optical and electrochemical characteristics, making their solid-state properties worthy of detailed investigation^12^. Furthermore, metal complexes of Et_2_DTC have demonstrated activity against various biological targets, emphasizing their potential for drug development as antibacterial, antioxidant and anticancer agents^4,13^. To the best of our knowledge, the present study represents one of the few systematic investigations that correlate the structural features of mono- and mixed-metal diethyldithiocarbamate complexes containing Ag, Cu, Mn, and selenium as an additional chalcogenide source. Moreover, the dielectric properties and alternating current (AC) conductivity of Et₂DTC and its metal complexes were investigated using a Keithley LCZ Meter Bridge to gain deeper insight into their electrical behavior and possible conduction mechanisms.

## Experimental

### Materials

All the chemical materials (sodium diethyldithiocarbamate trihydrate (NaEt_2_DTC.3H_2_O), CuCl_2_.2H_2_O, MnCl_2_.4H_2_O, AgNO_3,_ and NaHSeO_3_) and solvents (ethanol and acetonitrile) are supplied from Sigma-Aldrich (Germany) with good reagent grade for analytical uses.

### Synthesis of mononuclear complexes

**Fig. 1:**
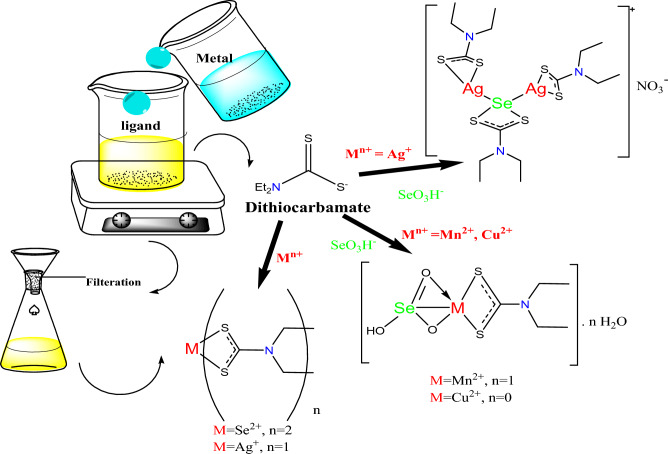
Schematic representation of synthetic procedure and proposed structures of complexes.

Silver diethyldithiocarbamate complex, [Ag(Et_2_DTC)], was prepared similarly as previously reported method^13^, where 3 mmol of AgNO_3_ salt was dissolved in distilled water. Ethanol was the solvent utilized to dissolve the required weight of 3 mmol of NaEt_2_DTC⋅3H_2_O. In case of [Se(Et_2_DTC)_2_] complex, the reaction was carried out using an excess of sodium diethyldithiocarbamate, which acts both as a coordinating ligand and as a mild reducing agent for the in-situ reduction of Se(IV) to a lower oxidation state Se(II)^14^. Drop by drop, the solution of metal has been added to the ligand while being stirred. Finally, all mixtures were left in a water bath for 30 min, then left overnight for complete precipitation. Filtration, solvent washing, and drying processes were performed on the produced mononuclear complexes. Figure 1 shows a schematic representation of the synthetic procedure and the proposed structures of complexes. The physical data of mononuclear complexes (elemental analysis, melting point, color, *μ*_eff_, *λ*_max_, and conductance) are listed in Table S1.

### Synthesis of mixed complexes

The required amount of NaEt_2_DTC.3H_2_O (3 mmol) was dissolved in 30 ml of ethanol and then mixed dropwise, under constant stirring, with 30 ml of an ethanolic solution of NaHSeO_3_ (3 mmol). The mixture gradually turned yellow. Subsequently, an ethanolic solution of metal salts [Mn (II), Cu (II), and Ag(I)] (3 mmol) was added dropwise to the mixture with continuous stirring in a water bath for 30 min. Likewise, in case of Ag-Se mixed complex, the amount of the ligand NaEt_2_DTC.3H_2_O was doubled, since part of the ligand acts as a redox partner and is consumed in the in-situ reduction of Se(IV) (from selenite) to Se(II) prior to chelation^14^.The mixture was then left overnight for complete precipitation. The resulting complexes were then filtered, given an ethanol wash, and allowed to dry. The suggested structure and the synthetic procedure for the mixed complexes are illustrated in Fig. 1. The physical data of the newly prepared mixed metal complexes are listed in Table S2.

### Instrumentation and methodology

CHNS contents were determined using a PerkinElmer elemental analyzer (model 240011). The melting points were recorded using a Stuart Scientific SMP 1 device and uncorrected. The FT-IR Bruker Tensor 37 was applied to record infrared spectra in the 400–4000 cm^−1^ spectral region utilizing the KBr disc method. Mass spectra were obtained at 70 eV by a Shimadzu QP-2010 plus mass spectrometer. H^1^ NMR and C^13^ NMR were recorded in DMSO solvent for ligand and diamagnetic complexes by Joel JNM ECZ500R NMR at 500 MHz and 11.75 Tesla. The XRD-D2 phaser was utilized to record X-ray diffraction for complexes using Cu radiation of wavelength value = 1.54184 Aͦ. EDX analysis and SEM imaging were obtained by SEM–EDX model [JEOL-JSM 5300]. In the 200–600 nm region, the UV–Visible spectra were recorded using a Lambda 4B Perkin-Elmer spectrophotometer model in acetonitrile solvent. All optical measurements were performed using a standard quartz cuvette with a 1 cm optical path length. The conductance was obtained at a concentration of 1 × 10^–3^ M in ethanol at room temperature, using a conductivity meter model HANNA (HI 8033) with a cell constant of 0.999 cm^−1^. At room temperature (305.5 K), magnetic susceptibility was recorded with a Sherwood model of scientific magnetic balance. The Perkin Elmer LS 55 fluorescence spectrophotometer was used to study the emission spectra of the prepared complexes in acetonitrile solvent with an excitation wavelength of 260 nm. The ESR spectrum of the copper-selenium mixed complex was determined via a JES-FE2XG-ESR device operating at 8.7 GHz. Magnetic hysteresis (M–H) loop of Mn-Se complex at room temperature was measured using a vibrating sample magnetometer (VSM) 7410 series. The measurements of AC (alternating current) impedance (Z) and capacitance (C) were carried out by using an AC Keithley LCZ Meter Bridge 3321, within frequencies values 120–10^5^ Hz and a temperature range from 296 up to 400 K. Different thermal analyses studies of the compounds were performed by a Bruker LINSEIS STA PT 1000 under N_2_ gas flow, with a heating rate of 10 °C min^–1^.

## Results and discussion

### FT-IR, NMR, and mass spectroscopic studies

**Fig. 2:**
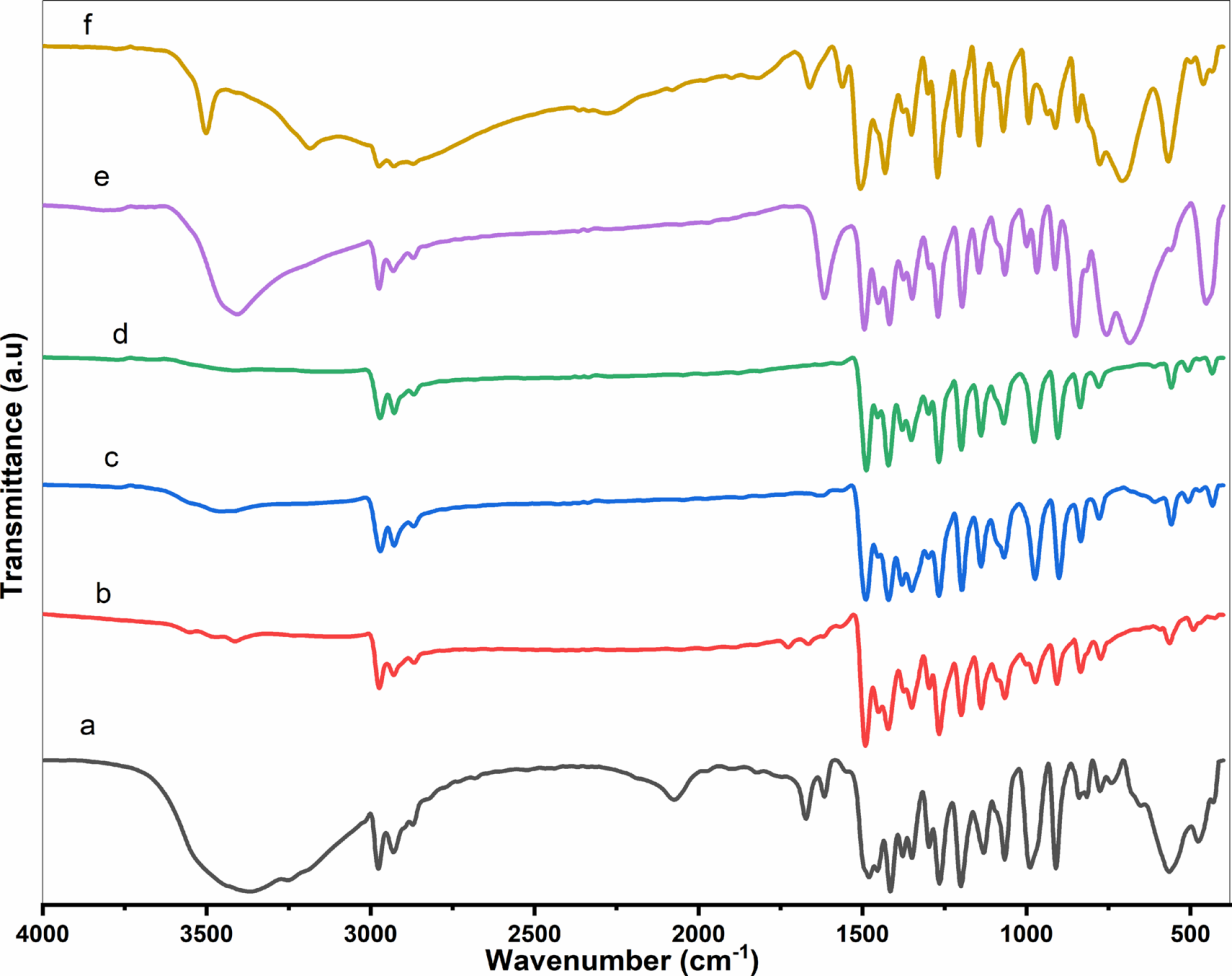
FTIR of investigated compounds; (a) Et_2_DTC; (b) [Se(Et_2_DTC)_2_]; (c) [Ag(Et_2_DTC)]; (d) [Ag_2_Se(Et_2_DTC)_3_]⋅NO_3_; (e) [MnSeO_3_H(Et_2_DTC)]⋅H_2_O; (f) [CuSeO_3_H(Et_2_DTC)].

The FTIR spectra of Et_2_DTC, [Se(Et_2_DTC)_2_], [Ag(Et_2_DTC)], [Ag_2_Se(Et_2_DTC)_3_].NO_3_ [MnSeO_3_H(Et_2_DTC)].H_2_O and [CuSeO_3_H(Et_2_DTC)] are given in Fig. 2 and Table S3, with some important characteristic assignments. The FTIR spectra showed that the ν (C–N) band of the thiocarbamate (NCS_2_) moiety at 1491, 1489, 1488, 1494, and 1506 cm^−1^ for [Se(Et_2_DTC)_2_], [Ag(Et_2_DTC)], [Ag_2_Se(Et_2_DTC)_3_].NO_3_, [MnSeO_3_H(Et_2_DTC)].H_2_O, and [CuSeO_3_H(Et_2_DTC)], respectively. This stretching vibration ν (C–N) in all complexes is obviously shifted to higher frequencies compared with that of the free ligand (Et_2_DTC) observed at 1480 cm^−1^. This shift can be attributed to the change in electron density upon coordination to the metal ion, indicating an increase in the bond order or a partial double bond character of C-N bond in the chelated ligand compared to its free form^4,13^. Another characteristic band associated with the coordination of the diethyldithiocarbamate ligand appears in the range 973–999 cm^−1^ corresponding to the ν (C–S) stretching vibration. The unsplit nature of this band suggests that the ligand (Et_2_DTC) coordinates in a bidentate manner via its two sulfur atoms^13^. In the [Ag_2_Se(Et_2_DTC)_3_].NO_3_ complex, two bands were observed at 1488 and 976 cm^−1^, characteristic of the bridging coordination mode of the Et_2_DTC ligand^15^.

The spectrum of the [MnSeO_3_H(Et_2_DTC)].H_2_O exhibits a broad band around 3406 cm^−1^ corresponding to ν (O–H) stretching vibration, along with a band at 1616 cm^−1^ of δ(O–H) bending, indicating the presence of hydrated water^13^. In case of [CuSeO_3_H(Et_2_DTC)], a sharp band at 3501 cm^−1^ is assigned to the existence of OH group of the hydrogen selenite (HSeO_3_^−^) moiety^15^. The relatively high wavenumber and sharp nature of this band (~ 3501 cm⁻^1^) indicate a weakly hydrogen-bonded O–H group, distinguishing it from the broader, lower-frequency O–H stretching band associated with lattice water. As well, [MnSeO_3_H(Et_2_DTC)].H_2_O and [CuSeO_3_H(Et_2_DTC)] complexes display two distinct bands at 755–684 cm^−1^ and 776–708 cm^−1^, respectively, which are assigned to Se-O vibrations modes of the hydrogen selenite (HSeO_3_⁻) group, indicating that selenium largely retains its + 4 oxidation state in these systems.^16^. However, these Se-O vibrations bands of HSeO_3_⁻ are absent in Se(Et_2_DTC)_2_ and [Ag_2_Se(Et_2_DTC)_3_].NO_3_ complexes suggesting the possible partial reduction of Se(IV) into Se(II) under the preparation reaction conditions. This difference may be attributed to the strong affinity of soft Ag(I) center toward soft selenium ions. Meanwhile, the harder metal Mn (II) and Cu (II) centers preferentially coordinate the oxygen atoms of HSeO_3_⁻ group in accordance with the HSAB principle, thereby stabilizing Se(IV). Similar coordination behavior has been reported for related Co (III) complexes containing hydrogen selenite ligands^16^. Furthermore, metal–ligand coordination is also indicated by the appearance of new bands in the 432–450 cm^−1^ region of the complexes, corresponding to M-S bond. These bands support the bidentate coordination of the NCS_2_ functional group of the Et_2_DTC with the metal ions (Se, Ag, Cu, and Mn)^15^. Figure 1 illustrates the proposed structures of the investigated complexes.

**Fig. 3.**
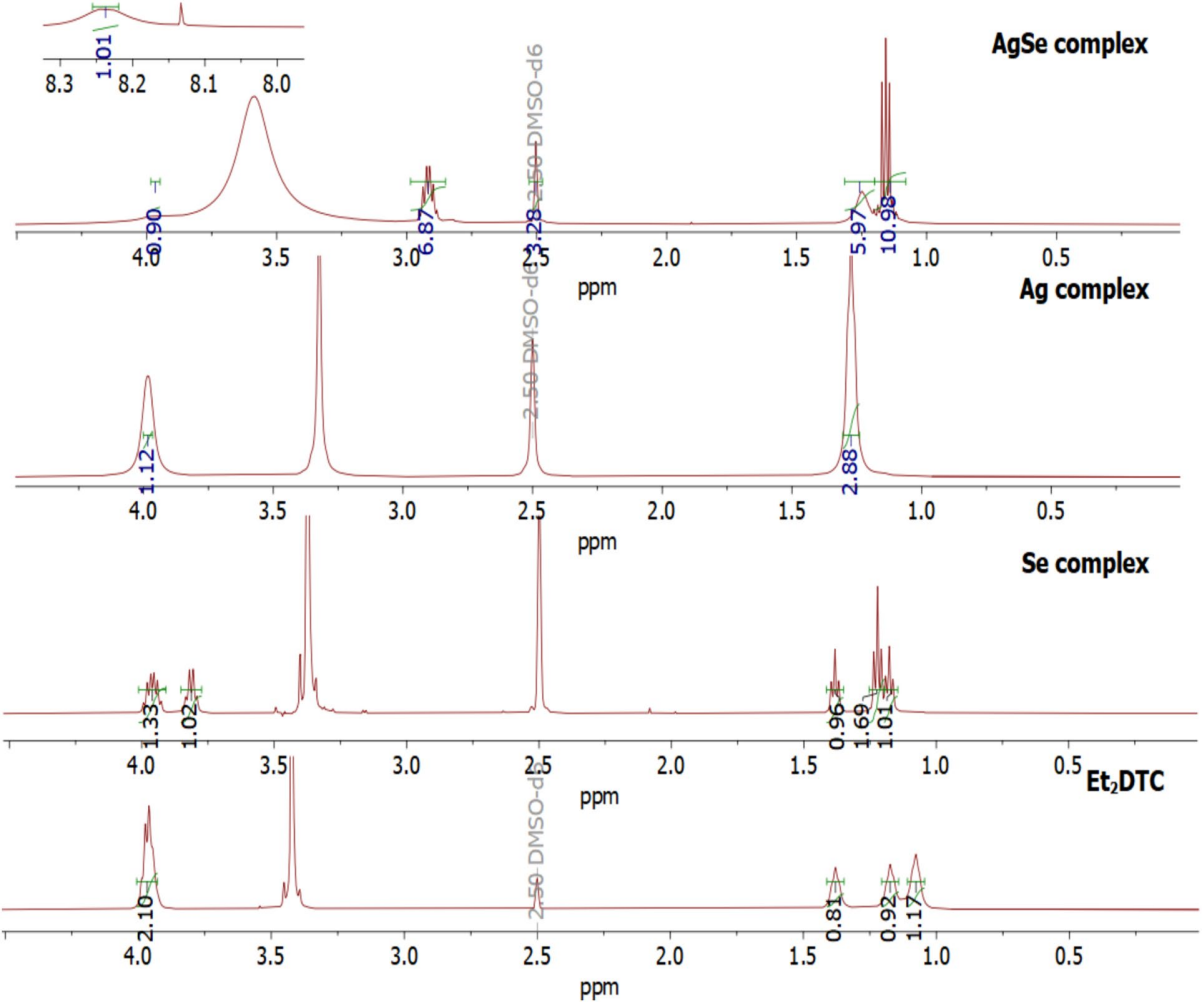
^1^H NMR of Et_2_DTC ligand and diamagnetic complexes.

In DMSO-*d*_6_, the ^1^H and ^13^C NMR spectra were obtained for diethyldithiocarbamate (Et_2_DTC) and diamagnetic complexes ([Se(Et_2_DTC)_2_], [Ag(Et_2_DTC)] and [Ag_2_Se(Et_2_DTC)_3_].NO_3_). Figure 3 shows the ^1^H NMR of the diamagnetic compounds. The protons of the (CH_2_) and (CH_3_) groups in the diethyldithiocarbamate (Et_2_DTC) appear as quartet and triplet peaks at chemical shift of δ 3.95–3.91 and δ 1.35–1.02 ppm, respectively^17^. The ^1^H NMR spectrum of [Se(Et_2_DTC)_2_] complex exhibits characteristic CH_3_CH_2_- signals at ranges: a three-triplet peak at 1.36–1.13 ppm of (CH_3_) groups and a two-peak for (CH_2_) groups, one of them a quartet at 3.80–3.76 ppm and a quintet at 3.96–3.89 ppm, respectively. This confirms the involvement of selenium in coordination and the chelation induced electronic effect on the ethyl groups of Et_2_DTC^18^.

The ^1^H NMR spectrum of [Ag(Et_2_DTC)] complex shows a signal for the (CH_2_) group at δ 3.95 ppm, along with an upfield shift of (CH_3_) groups at δ 1.48–0.96 ppm. This indicates that the long-range chemical shielding of the ethyl group is only slightly affected by the geometrical variations of the S atoms in Et_2_DTC ligand^19^. The ^1^H NMR spectrum of [Ag_2_Se(Et_2_DTC)_3_].NO_3_ displays three peaks: a triplet peak within δ (1.30–1.10) ppm due to the methyl group^18^, a quintet peak within δ (2.95–2.79) ppm due to the methylene group^19^, and the third peak within δ (8.24–8.13) ppm. The latter peak was observed because of the nuclear Overhauser effect between protons^20^ and the distinct proton environments in the diethyldithiocarbamate upon complexation with two hetero-metal ions in [Ag_2_Se(Et_2_DTC)_3_].NO_3_^13,20^.

Figure S1 shows the ^13^C NMR of diethyldithiocarbamate and the examined complexes. The ^13^C NMR spectra of Et_2_DTC confirms the complex formation and suggested structures of [Se(Et_2_DTC)_2_], [Ag(Et_2_DTC)] and [Ag_2_Se(Et_2_DTC)_3_].NO_3_. The spectra of diethyldithiocarbamate and its diamagnetic complexes showed a characteristic peak at δ (190.56–209.84) ppm for the carbon of the (NCS_2_) group. Whereas the chemical shift of the carbon atom of methyl and methylene groups of the Et_2_DTC ligand appeared at δ (10.93–13.20) ppm and δ (41.65–51.57) ppm, respectively^18^. The upfield shift was observed for the methylene carbon of [Ag_2_Se(Et_2_DTC)_3_].NO_3_ at δ (41.65) ppm. This confirms the upfield of the quintet peak of the CH_2_ group in ^1^H NMR.

The mass spectra data (Fig. S2) of the mixed complexes [MnSeO_3_H(Et_2_DTC)].H_2_O, [CuSeO_3_H(Et_2_DTC)], and [Ag_2_Se(Et_2_DTC)_3_].NO_3_ showed molecular ion peak (M^+•^) at *m/z* = 354, 355, and 789.2, respectively, which sufficiently correlate well with their formula weights and also support their chemical structures as determined by various spectral and analytical methods. Also, the appearance of metal isotopes (Se, Ag, Cu, and Mn) near their *m/z* value supports the complexation process. The parent ion was at *m/z* 116 (C_5_H_10_NS) for Mn-Se complex and Cu-Se complex, whereas for Ag-Se complex, it was at *m/z* 58 (C_3_H_8_N). An additional peak at *m/z* 359 was detected in the spectrum of all samples. This peak likely arises from a fragmentation byproduct corresponding to disulfiram, which is formed from the combination of two Et₂DTC radicals during ionization^21^.

### X-ray powder diffraction

**Fig. 4:**
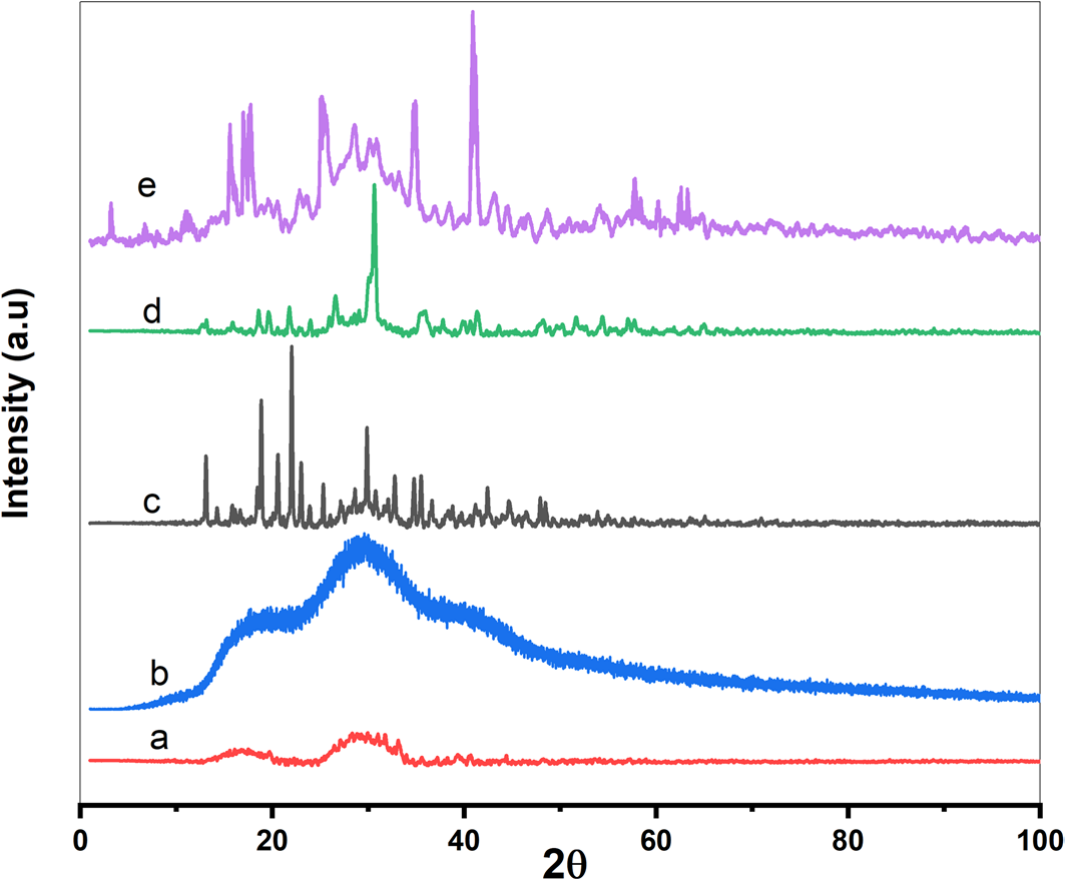
Powder XRD graph of (a) [Ag(Et_2_DTC)] (b) [Ag_2_Se(Et_2_DTC)_3_]⋅NO_3_ (c) [Se(Et_2_DTC)_2_] (d) [MnSeO_3_H(Et_2_DTC)]⋅H_2_O (e)[CuSeO_3_H(Et_2_DTC)].

Figure 4 displays the powder X-ray diffraction pattern for the metal complexes under investigation. The sharp peaks in the XRD pattern confirm the polycrystalline nature of the investigated complexes. The broad peaks in XRD spectra may be attributed to the disordering of crystal lattices and the indiscriminate orientation of atoms inside the 3D space^22^. The average size of the crystallite (*D*) was calculated utilizing the Scherrer formula^22^ as follows:

1 $$D= 0.9 \lambda /{\beta}_{hkl} cos\theta$$

*θ* stands for the Bragg diffraction angle, *λ* for the wavelength of X-ray (*λ* = 1.54184 Aͦ), and *β*_*hkl*_ for the full width at half maximum of the peak in radians. The calculated *D* values were 30.76, 15.43, 17.77, 0.66, and 1.19 nm for [Se(Et_2_DTC)_2_], [MnSeO_3_H(Et_2_DTC)].H_2_O, [CuSeO_3_H(Et_2_DTC)], [Ag(Et_2_DTC)], and [Ag_2_Se(Et_2_DTC)_3_].NO_3_ complexes, respectively, indicating that all the synthesized complexes crystallize on a nanoscale. These values provide an approximate indication of the coherent diffraction domain sizes and should be regarded as qualitative estimates rather than precise particle dimensions. The small particle sizes of these complexes suggest that diethyldithiocarbamates can form 1D coordination polymers and multinuclear aggregates^23^.

Furthermore, the Williamson-Hall equation was applied as an empirical method to qualitatively separate the contributions of crystallite size and strain-like broadening to the powder XRD peak profiles of the diethyldithiocarbamate complexes^24^:

2 $${\beta}_{hkl} cos{\theta}_{hkl}= 0.9\lambda /D+4\varepsilon sin{\theta}_{hkl}$$

where *D* represents the average size of crystallite and *ε* is a phenomenological strain parameter. The W–H curves of diethyldithiocarbamate complexes were constructed by plotting *β*_*hkl*_ cosθ_hkl_ versus 4sinθ_hkl_ using five or three intense diffraction peaks. Table S4 includes the values of *ε*, and* D* of the isolated complexes based on slope and interception, as illustrated in Fig. S3. As commonly observed, the crystallite size (*D*) values obtained by the W–H model are generally larger than those calculated by Scherrer’s equation, reflecting the inclusion of strain-like broadening effects in the W–H analysis^24^. It should be emphasized that, for molecular coordination complexes, the ε values should be regarded as qualitative indicators of peak broadening rather than as true measures of elastic lattice strain.

The number of dislocations in a unit volume of a crystalline material is measured by the dislocation density, which is a crystallographic defect or imperfection inside a crystal structure. The dislocation density ($\delta$) for metal complexes is calculated as follows^22^:

3 $$\delta = 1/{D}^{2}$$

where *D* is determined using Scherrer’s formula. The calculated *δ* values were 0.001 × 10^12^, 0.004 × 10^12^, 0.003 × 10^12^, 2.313 × 10^12^ and 0.709 × 10^12^ mm^−2^ for [Se(Et_2_DTC)_2_], [MnSeO_3_H(Et_2_DTC)].H_2_O, [CuSeO_3_H(Et_2_DTC)], [Ag(Et_2_DTC)] and [Ag_2_Se(Et_2_DTC)_3_].NO_3_ complexes, respectively. Since *D* values derived from Scherrer analysis represent approximate crystallite dimensions and assume isotropic peak broadening, the corresponding δ values should be regarded as comparative rather than absolute measures of lattice defects. The relatively higher δ values observed for the silver-containing complexes ([Ag(Et_2_DTC)] and Ag-Se) suggest increased microstructural disorder or smaller apparent crystallite size, whereas lower δ values for the [Se(Et_2_DTC)_2_], Mn–Se, and Cu–Se complexes indicate comparatively higher crystallinity^22^. To further support this analysis, the degree of crystallinity (*X*_*c*_) was estimated using Eq. (4), by integrating the area under the crystalline diffraction peaks relative to the total diffracted area^25^:

4 $${x}_{c}=\frac{{A}_{c}}{{A}_{c}+{A}_{a}}\cdot 100$$

where the *A*_*c*_ is the area under crystalline peak and *A*_*a*_ represents the area under amorphous peaks. The estimated crystallinity values range from 50.9% to 95.4%. The Ag-containing complexes, [Ag(Et₂DTC)] and [Ag₂Se(Et₂DTC)₃]NO₃, exhibit the lowest crystallinity values (62.7% and 50.9%, respectively), which is consistent with their higher δ values and broader diffraction peaks (Fig. 4 a, b). In contrast, [Se(Et₂DTC)₂], Mn–Se, and Cu–Se complexes show higher crystallinity values of 70.2%, 84.0%, and 95.4%, respectively, suggesting comparatively better structural ordering. Variation in crystallinity may influence the optical and electrical properties of these materials; higher crystallinity is generally associated with enhanced charge transport pathways and improved thermal stability, whereas lower crystallinity may increase defect density and surface-related activity. XRD analysis revealed that the Se(Et_2_DTC)_2_] and Mn-Se complexes crystallize in orthorhombic system, whereas the [Ag(Et_2_DTC)] and Cu-Se complexes adopt a monoclinic structure. In contrast, Ag-Se complex is indexed to a cubic crystal system. Table S4 presents the crystal system, lattice parameters, and space group, and unit cell volume for the polycrystalline complexes.

### Microstructural analysis and surface morphology

**Fig. 5:**
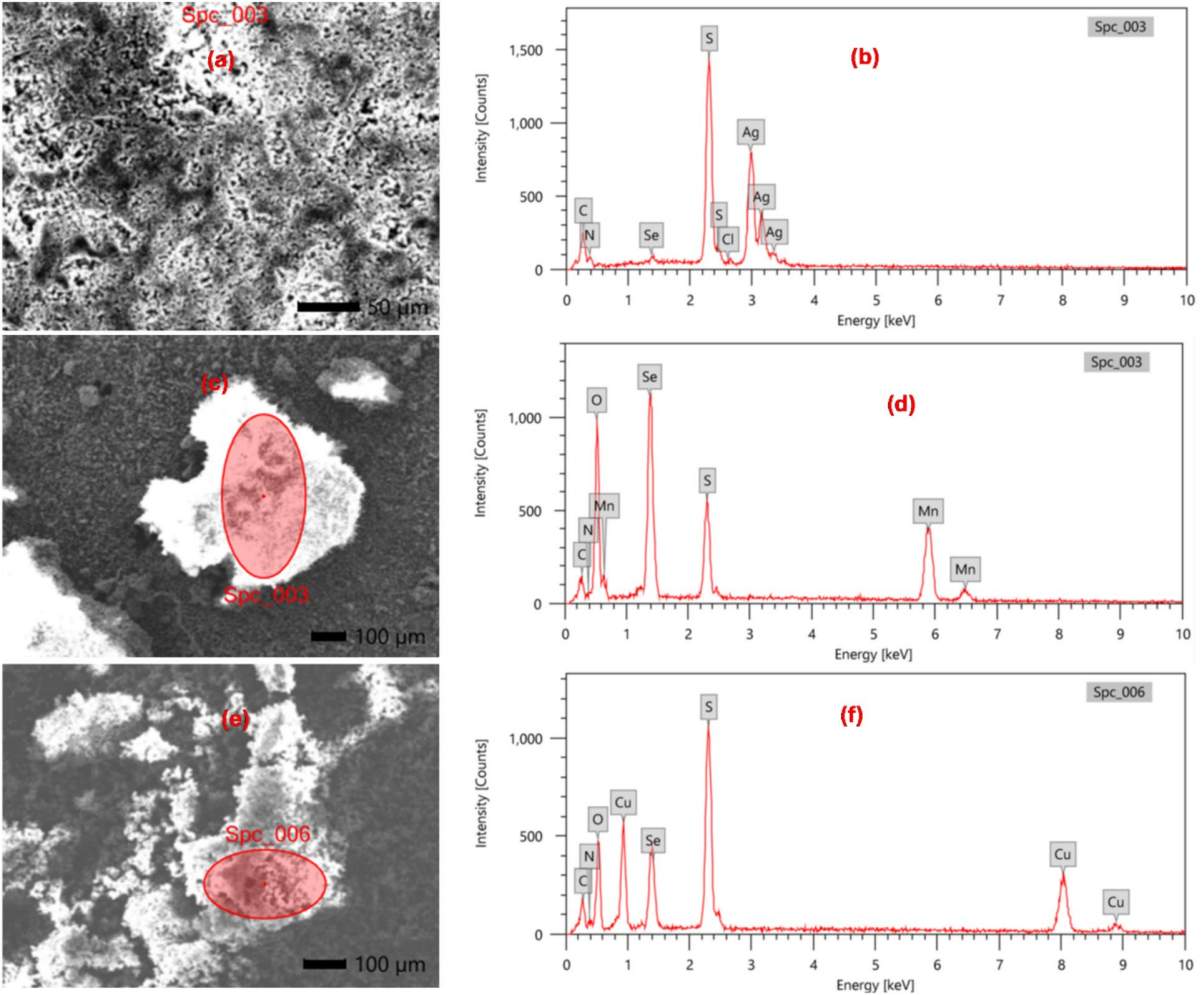
SEM and EDX analysis for; (**a**, **b**) [Ag_2_Se(Et_2_DTC)_3_]⋅NO_3_; (**c**, **d**) [MnSeO_3_H(Et_2_DTC)]⋅H_2_O; (**e**, **f**) [CuSeO_3_H(Et_2_DTC)].

Figure 5a, c, e and Fig. S4a, c display the surface morphologies of the investigated complexes. The SEM photographs reveal non-uniform particle size distributions, irregular aggregates, and differences in morphology. The [Se(Et_2_DTC)_2_] complex shows a hexagonal structure, confirming its crystalline, homogeneous nature^26^. In contrast, the other complexes exhibit a sponge-like structure resembling a ball microsphere^27^. The EDX analysis provides information on the % atomic abundance of elements on the surface of each sample enabling the assessment of chemical stoichiometry and elemental distribution of the metal complexes^28^. Figure 5b, d, f and Fig. S4b, d show EDX spectra of the synthesised metal complexes. The atomic ratios between the constituent elements of the ligand and the metal ions confirm the chelating ability of diethyldithiocarbamate to form assembled and hierarchical network structures with metal ion^27^. Also, the EDX analysis further supports the reducing ability of diethyldithiocarbamate toward biselenite, as evidenced by the absence of oxygen peak in the spectra of [Se(Et_2_DTC)_2_] and Ag-Se complexes^19^. Although nitrate is present as a counterion in [Ag₂Se(Et₂DTC)₃].NO_3_, its relatively low abundance and weak association with the coordination sphere, combined with the surface-sensitive and semi-quantitative nature of EDX, may account for the absence or very weak intensity of an oxygen peak^28^. Conversely, in the [MnSeO_3_H(Et_2_DTC)].H_2_O and [CuSeO_3_H(Et_2_DTC)] complexes, a distinct oxygen peak of the biselenite moiety is clearly observed, consistent with the presence of structurally bound biselenite group. These observations are therefore in agreement with the reduction of Se(IV) to Se(II) in the former complexes and the retention of Se(IV) in the latter. The [Ag(Et_2_DTC)] complex shows a small peak corresponding to oxygen atom, confirming the porosity of the surface and hence oxidation of the exposed product upon atmospheric contact, consistent with the high surface-to-volume ratio of typical silver complexes^27^. So, this analysis supported the purity of the synthesized metal complexes^26,27^.

### Electronic spectra, magnetic, and molar conductance measurements

**Fig. 6:**
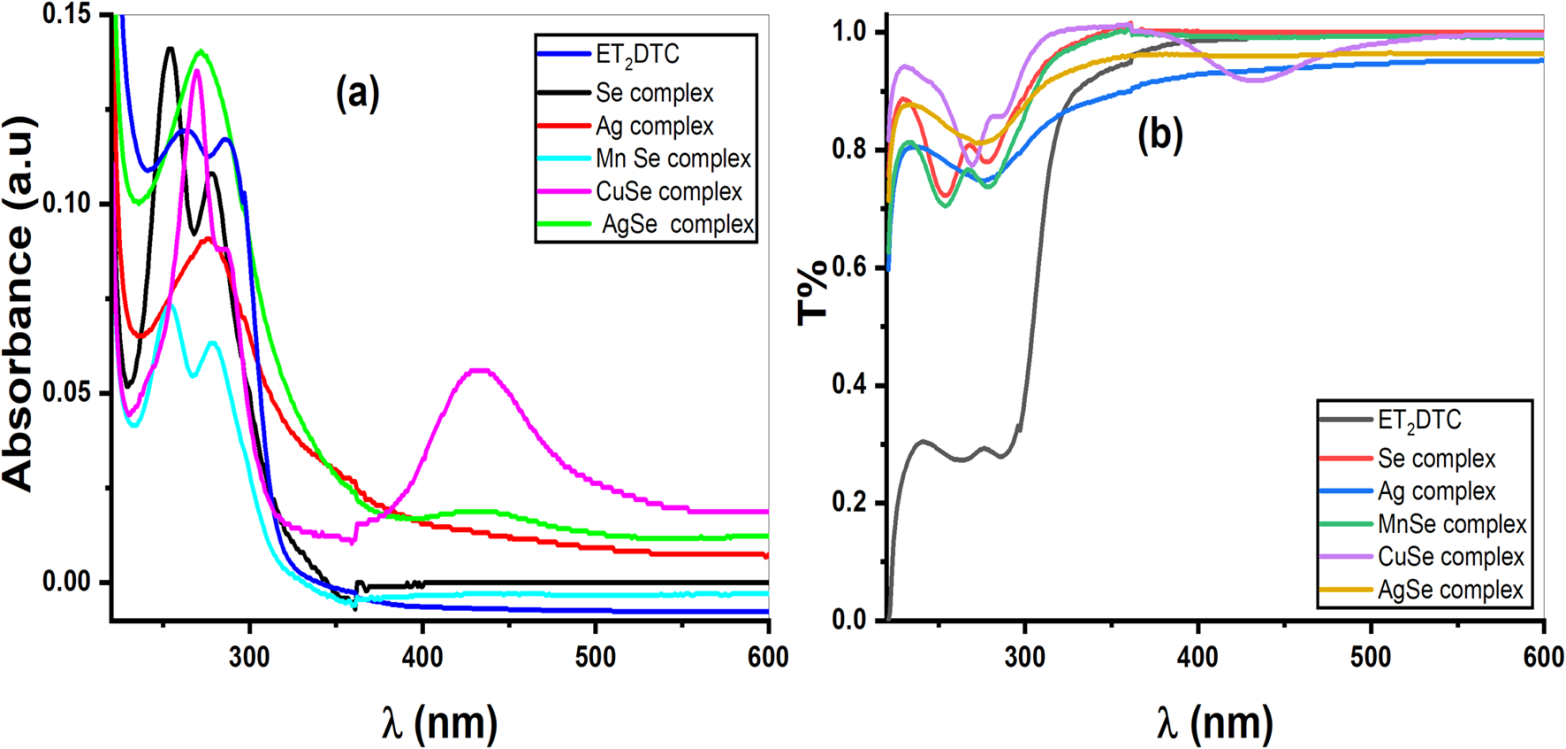
UV–Visible spectra of complexes based on diethyldithiocarbamate (**a**) variation of absorption with wavelength (*λ*) (**b**) relation between transmission (T%) and wavelength (*λ*).

The UV–Visible absorption spectra of the ligand (Et_2_DTC) and its five compounds in acetonitrile solvent are shown in Fig. 6a. The values of *μ*_eff_ and *λ*_max_ of the metal complexes are listed in Tables S1 and S2. The high energy absorption bands in Et_2_DTC ligand at 260 and 285 nm are due to the transitions from *n* or *π* orbitals to π* orbital within the C-N or C = S chromophores, as well as intra ligand charge transfer transitions (ILCT) or ligand to ligand charge transfer (LLCT) transitions^23,29^. These bands are observed in the metal complexes but shifted to lower wavelength (blue shift) due to the participation of NCS_2_ in complex formation. These transitions are characteristic of a typical tetrahedral geometry for the high spin [MnSeO_3_H(Et_2_DTC)].H_2_O and the diamagnetic [Se(Et_2_DTC)_2_]^13^. For the [Ag_2_Se(Et_2_DTC)_3_].NO_3_ complex, the ligand-to-metal charge transfer (LMCT) is responsible for the higher wavelength maximum at 438 nm^19,30^. Likewise, the [CuSeO_3_H(Et_2_DTC)] exhibits a broad band at 433 nm because of the transfer of charge from ligand to copper ion. The broad nature of the latter band likely masks the d-d transition associated with square planar shape of the copper (II) center^13,29^.

The magnetic susceptibility values (*μ*_eff_) at 305.5 K of the mononuclear and mixed complexes are listed in Tables S1 and S2, respectively. The complexes of [Se(Et_2_DTC)_2_], [Ag(Et_2_DTC)] [Ag_2_Se(Et_2_DTC)_3_].NO_3_ exhibit diamagnetic behavior. While isolated Se(II) ions are formally paramagnetic (4p^2^ configuration), the ligand field in the chelated complexes can pair the 4p electrons, resulting in the observed diamagnetism. However, the mixed complexes of Mn-Se and Cu-Se are paramagnetic and have *μ*_eff_ 7.23 and 2.52 B.M, respectively. These complexes have a magnetic moment higher than the spin-only value due to orbital contribution and spin–orbit coupling with the selenium atom^13^. Also, this confirms the participation of the biselenite anion in chelation and the presence of Se(IV) that increases the value of the magnetic moments of these complexes. The mixed complexes of Mn-Se and Cu-Se have a tetrahedral geometry. Tables S1 and S2 summarize the molar conductance (*Λ*_m_) of the mononuclear and mixed complexes. Most of the complexes exhibit as non-electrolyte with very low values of *Λ*_m_ (1.89–3.82 Ω^−1^ mol^−1^cm^2^). In contrast, [Ag_2_Se(Et_2_DTC)_3_].NO_3_ shows a significantly higher *Λ*_m_ value of 50 Ω^−1^ mol^−1^cm^2^ in ethanol, confirming its electrolytic nature due to the presence of the nitrate ion in the outer sphere.

### Optical analysis

**Fig. 7:**
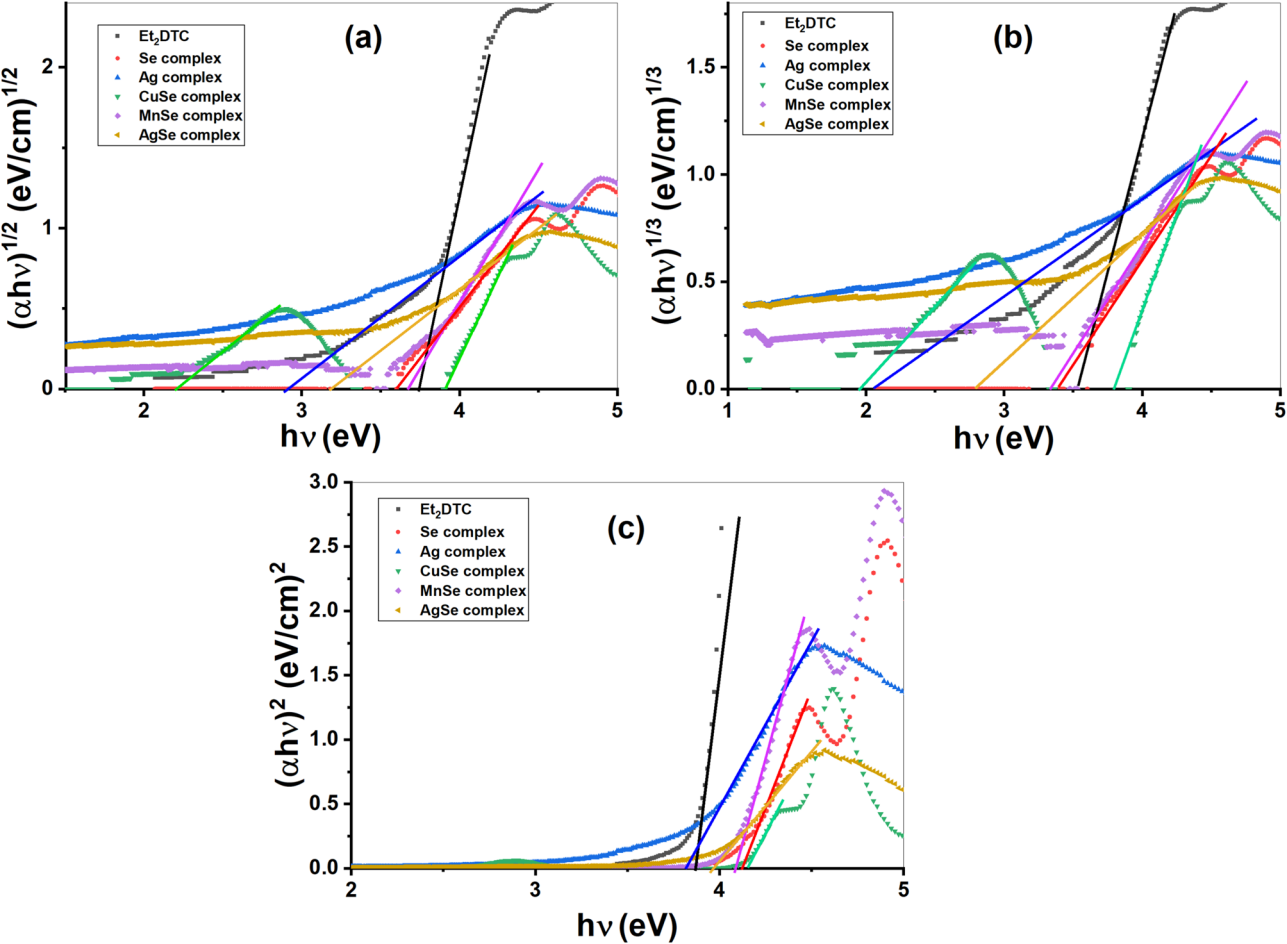
Plot of photon energy (hν) against (**a**) (αhν)^1/2^ for indirect allowed bandgap (**b**) (αhν)^1/3^ for indirect forbidden bandgap (**c**) (αhν)^2^ for direct allowed bandgap.

The transmittance of diethyldithiocarbamate and its based complexes is displayed in Fig. 6b. The transmission increases gradually at wavelength around 322 nm and with high percentage about 86%, 94%, 84%, 94%, 99%, 90% for Et_2_DTC, [Se(Et_2_DTC)_2_], [Ag(Et_2_DTC)], [MnSeO_3_H(Et_2_DTC)].H_2_O, [CuSeO_3_H(Et_2_DTC)] and [Ag_2_Se(Et_2_DTC)_3_].NO_3_, respectively. Thus, the diethyldithiocarbamate ligand and its metal-based complexes display optically transparency above approximately 322 nm. The Tauc equation was utilized to determine the optical band gap (*E*_*g*_) of the complexes^31,32^:

5 $$\alpha h\nu = {B(h\nu -{E}_{g})}^{n}$$

where *α* is absorption coefficient and equals 2.303(*A/l*) where (*A*) representing the absorbance and (*l*) the optical path length (1 cm). *B* and *v* are a Tauc constant and frequency, respectively. However, the exponent *n* is dependent on the nature of electronic transitions occurring in the semiconductors. For direct and indirect allowed transitions, *n* = ½ and 2, respectively, while for direct and indirect forbidden transitions, *n* = 3/2 and 3, respectively^31,32^. Figure 7a, b, and c show the Tauc relation for indirect (allowed and forbidden) and direct allowed bandgap, respectively. Table 1 lists the determined optical band gap values (*E*_g1_, *E*_g2_, *E*_g3_) indirect (allowed, forbidden) and direct allowed for the examined compounds, respectively. These values range between 1.95 and 4.15 eV and are relatively close to each other in all applied models in Fig. 7.

**Table 1:** Optical characteristics of Et_2_DTC and its complexes.

Compound	Et_2_DTC	[Se(Et_2_DTC)_2_]	[Ag(Et_2_DTC)]	[Ag_2_Se(Et_2_DTC)_3_]⋅NO_3_	[CuSeO_3_H(Et_2_DTC)]	[MnSeO_3_H(Et_2_DTC)]⋅H_2_O
E_g1_ (eV)	3.74	3.6	2.9	3.18	2.21, 3.92	3.67
E_g2_ (eV)	3.52	3.39	2.04	2.79	1.95, 3.79	3.39
E_g3_ (eV)	3.86	4.12	3.80	3.96	4.15	4.08
Sₒ (nm^2^)	4.32E − 06	1.15E − 06	1.31E − 05	5.19E − 06	5.83E − 07	1.09E − 06
λₒ (nm)	289.90	291.68	249.89	267.88	290.52	294.45
Eₒ (eV)	4.30	4.22	4.98	4.65	4.25	4.21
Ed (eV)	1.58	0.32	4.07	1.74	0.15	0.35
F (eV)^2^	6.78	1.36	20.26	8.07	0.62	1.49
εₒ	3.72	14.08	2.22	3.67	30.12	12.88
nₒ	1.93	3.75	1.49	1.92	5.49	3.59
M_−1_	0.37	0.08	0.82	0.37	0.03	0.08
M_−3_ (eV)^−2^	1.99E − 02	4.29E − 03	3.30E − 02	1.73E − 02	1.90E − 03	4.76E − 03
N/m* (kg^−1^ m^−3^)	5.34E + 41	7.62E + 40	5.45E + 40	5.36E + 40	9.35E + 40	1.05E + 41
ε_∞_	47.19	8.06	7.60	6.68	8.74	10.45

**Fig. 8:**
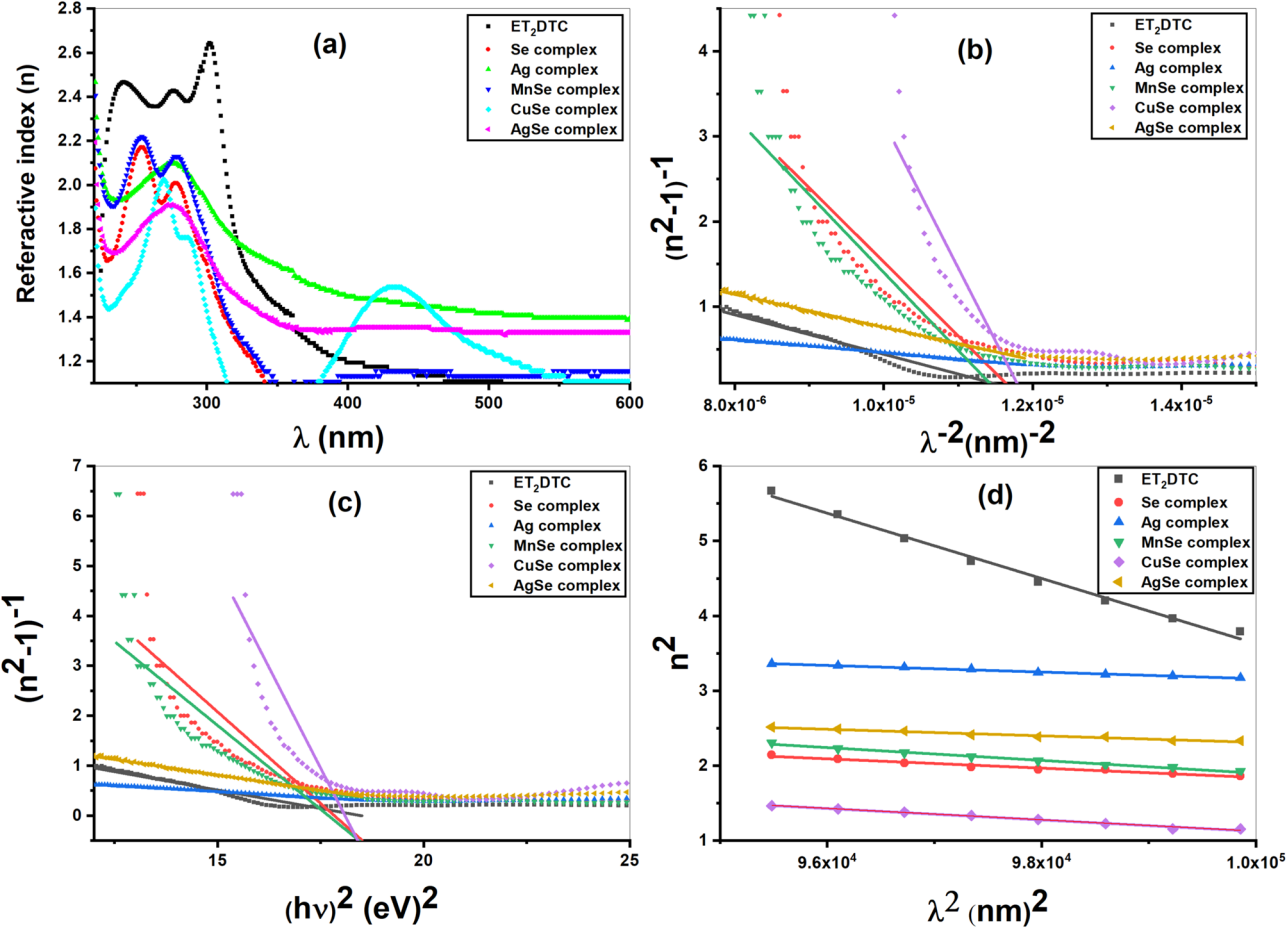
(**a**) Variation of *n* with* λ*, (**b**) plot of *(n*^*2*^* − 1)*^*−1*^ against *λ*^*−2*^, (**c**) plot of *(n*^*2*^* − 1)*^*−1*^ vs, *(hν)*^*2*^, (**d**) variation between *n*^*2*^ and *λ*^*2*^ for ET_2_DTC and its complexes.

The refractive index (*n*) of substance is an important optical parameter that reflects its interaction with electromagnetic radiation and is related to its electronic polarizability. The refractive index values for the investigated compounds were calculated using the approximate method based on simultaneous equations as described in the literature^33^. Figure 8a illustrates the variation of *n* versus *λ* for all samples. At shorter wavelength (*λ* ≈ 240–440 nm), the higher values of *n* are attributed to charge-transfer transitions occurring between energy levels within the complexes^23,29^. The multi-oscillator model was utilized to analyse this dissimilar dispersion^34^. The value of *n* dropped as *λ* increased at higher wavelengths (*λ* > 440 nm). According to the single-oscillator model, this indicates a common dispersion within this *λ* range^35^.

In the lower frequency range, the refractive index is given using Sellmeier’s classical dispersion model as follows^36^:

6 $${({n}^{2}-1)}^{-1}=1/{s}_{o}{{\lambda}_{o}}^{2}-1/{s}_{o}{\lambda }^{2}$$

7 $${s}_{o}= ({n}_{0}^{2}-1)/{{\lambda}_{o}}^{2}$$

where *s*_o_ and *λ*_o_ represent the oscillator’s strength and wavelength, respectively. At higher *λ*, Eq. (7) yields the static refractive index (*n*_o_). Figure 8b shows the variation of (*n*^2^ − 1)^−1^ against *λ*^−2^. Table 1 displays the values derived from Sellmeier’s model. It is observed that the oscillator wavelength and oscillator strength are inversely related to each other. The highest value of the oscillator strength (*s)*_o_ is 1.31 × 10^–5^ nm^2^ at oscillator wavelength (*λ*_o_) equal to 249.89 nm for [Ag(Et_2_DTC)] complex.

The main purpose in this part is to determine the optical parameters of the investigated materials. Also, theoretical models such as the single oscillator Wemple–DiDomenico model were applied to describe the dispersion behaviour of the linear refractive index, including the oscillator and dispersion energy. The dispersion of *n* is studied utilizing the Wemple and DiDomenico model, which yields the single oscillator energy *E*_o_ and the dispersion energy *E*_*d*_ as follows^35^:

8 $${({n}^{2}-1)}^{-1}=({E}_{o}/{E}_{d})-( 1/{E}_{o}{E}_{d}) {(h\nu )}^{2}$$

Figure 8c displays the plot of (*n*^2^ − 1)^−1^ against (*hv*)^2^, where the slope and intercept were used to obtain the values of *E*_o_ and *E*_*d*_, as listed in Table 1 The dispersion energy values reveal that the [Ag(Et_2_DTC)] complex has the highest value, which can be attributed to the oscillator strength of the band-to-band transition increases^37^.

Additionally, Table 1 lists the oscillator strength (*f* = *E*_*o*_*E*_*d*_*)* derived from the dispersion energy^36^, further confirming the high value of *E*_*d*_ for [Ag(Et_2_DTC)]. Moreover, the static refractive index (*n*_o_), zero frequency dielectric constant (*ε*_o_) and the first and the second optical moment spectra (*M*_*-1,*_ and *M*_*-3*_.) were obtained from the *E*_o_ and *E*_*d*_. The above parameters were determined as follows^37^.

9 $${\varepsilon}_{0} ={{n}_{o}}^{2} = 1+{(E}_{d}/{E}_{o})$$

10 $${M}_{-1}={E}_{d}/{E}_{o} \mathrm{a}\mathrm{n}\mathrm{d} { M}_{-3}= {M}_{-1}/{{E}_{o}}^{2}$$

Table 1 lists the estimated values of the parameters mentioned above, which exhibit a straight proportionality between *n*_o_ and *ε*_o_. Notably, *M*_*-1*_ values are greater than the values of *M*_*-3*_. Evaluating the optical characteristics of the samples under study requires determining the high-frequency dielectric constant (*ε*_*∞*_) and the carrier concentration per effective mass (N/m*). These parameters were obtained as follows^36,37^:

11 $${n}^{2} = {\varepsilon}_{\infty } - \left(1/4{\pi }^{2 }{\varepsilon}_{o}\right)\left(\frac{{e}^{2}}{{c}^{2}}\right)\left(\frac{N}{{m}^{*}}\right){\lambda }^{2}$$

where *ε*_o_ is the permittivity at vacuum (8.85 × 10^–12^ F/m), *e* is the charge of an electron (1.6 × 10^–19^ C), and *c* is the speed of light^37^. Figure 8d displays the graph of *n*^*2*^ versus *λ*^*2*^, where the slope and intercept were used to determine the value of N/m* and *ε*_*∞*_, respectively, as listed in Table 1. The estimated (*ε*_*∞*_) values increase while the carrier concentration decreases. Moreover, the *ε*_*∞*_ values are higher than *ε*_o_ (calculated by the Wemple-DiDomenico model) for some of the investigated complexes, except [Se(Et_2_DTC)_2_], [MnSeO_3_H(Et_2_DTC)].H_2_O, and [CuSeO_3_H(Et_2_DTC)], owing to the minor contribution of free carriers^12^.

### Fluorescence emission spectra

**Fig. 9:**
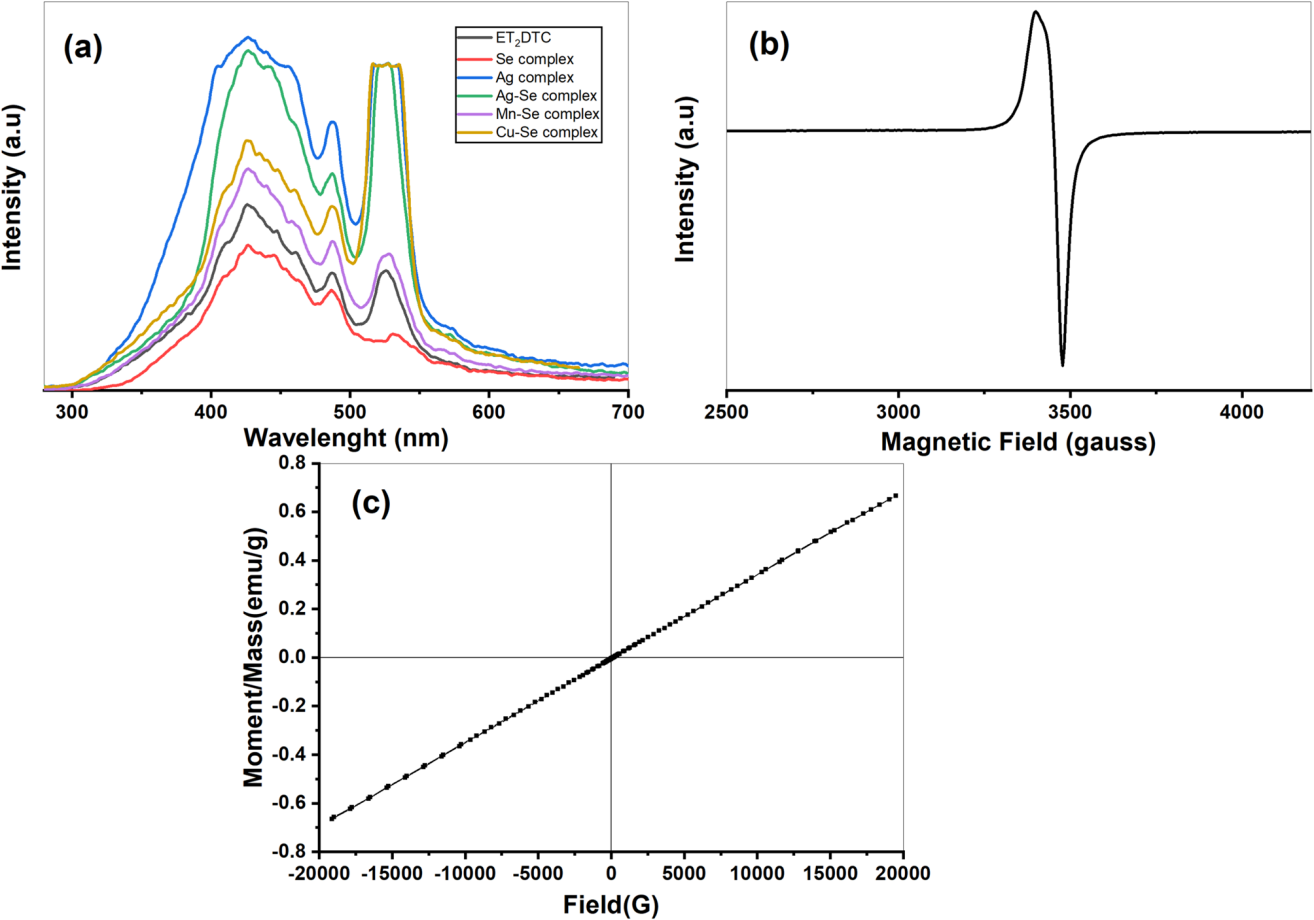
(**a**) Fluorescence emission spectra of investigated compound at *λ*_*ex*_ = 260 nm, (**b**) ESR spectra for [CuSeO_3_H(Et_2_DTC)], (**c**) Magnetic hysteresis of [MnSeO_3_H(Et_2_DTC)]⋅H_2_O.

Figure 9a shows the emission spectra of the Et_2_DTC ligand and its five complexes. The fluorescence emission spectra of investigated compounds upon excitation at *λ*_ex_ = 260 nm shows three emission peaks at 427, 487 nm, and a third at 527–531 nm. These emission bands are attributed to charge transfer from metal to diethyldithiocarbamate or vice versa^23^.

### Electron spin resonance (ESR)

Figure 9b shows the ESR spectrum of [CuSeO_3_H(Et_2_DTC)] at room temperature with a distinct spectral peak at magnetic field of *B*_°_ = 3450.48 gauss. The hyperfine coupling constant (*A* = 38.31 × 10^–4^ cm^−1^) and the computed splitting factor (*g*) for [CuSeO_3_H(Et_*2*_DTC)] equal 2.044, indicate isotropic spectral characteristics, which are typically associated with Cu (II) complexes with a lower symmetry than octahedral geometry^38^. The covalent bond character of *d*_*x*_^*2*^_*-y*_^*2*^ (*α*^*2*^) can be measured using the related isotropic *A* equation^38^:

12 $${\alpha }^{2}=\frac{\left|A\right|}{PK}+\frac{g-2.0023}{K}$$

where *P* and *K* represent the free-ion dipole (0.036 cm^−1^) and the Fermi contact (0.43), respectively. The contribution fraction of 4 s from apical ligands in the ground state (*f*^*2*^) is the cause of the assumption error in the equation mentioned above. So, Eq. (12) can be expressed as follows:

13 $${\alpha }^{2}=\frac{\left|A\right|}{PK}+\frac{g-2.0023}{K}+\frac{1-{f}^{2}}{PK}$$

For [CuSeO_3_H(Et_2_DTC)] complex, the computed values of *α*^*2*^ and *f*^*2*^ are 0.34445 and 0.01548, respectively^38^, based on the previously mentioned data. This confirms the participation of the *d*_*x*_^*2*^_*-y*_^*2*^ orbital, as indicated by *α*^*2*^ value less than 1, and suggest that the complex adopts a tetrahedral geometry.

### Magnetic hysteresis

Figure 9c describes the plot of magnetization against the magnetic field (M–H hysteresis loop)^39^ of [MnSeO_3_H(Et_2_DTC)].H_2_O at room temperature. The M-H curve of the Mn-Se complex has a paramagnetic susceptibility slope of 3.455 × 10^–5^. A positive linear magnetization response was observed when a magnetic field was applied to the [MnSeO_3_H(Et_2_DTC)].H_2_O complex, because of the high-spin character of the d orbital of Mn (II). The magnetization at maximum magnetic field (*M*_max_) for the Mn-Se complex was found to be 0.6657 emu/g. So, the mixed Mn-Se complex may become ferromagnetic below its Curie temperature^40^.

### Electrical measurements

Electrical studies were performed utilizing alternating current (AC) at various frequencies and temperatures for the ligand (Et_2_DTC), and its metal complexes.

#### Conductivity study

Equation (14) is used to compute the AC conductivity *σ (ω)*:

14 $$\sigma \left(\omega \right)=d/Z A$$

where *Z* is the measured resistance of the sample, *d* and *A* are its dimensions^12^. Fig. S5 illustrates the plot of *σ* (ω) versus *T* for the compounds at frequencies between 120 Hz and 100 kHz. For the present samples, *σ (ω)* versus *T* exhibits various conduction behaviors as the temperature increases, with *σ (ω)* values ranging from 10^–7^ to 10^–1^ *S/m*. Their relatively low conductivities may be attributed to weak interactions between sulfur atoms between the packed layers, as indicated by short S…S separations in the solid state^29^. Within the relevant temperature region, the complexes exhibit semiconductor-like behavior because of the positive relationship between *T* and *σ (ω)*. Different conduction regions were observed due to the presence of more than one conduction mechanism with temperature elevation^41^. The activation energy (*E*) is commonly expressed using the Arrhenius equation^42^:

15 $$\sigma = {\sigma}_{0} exp(-E/ {k}_{B}T)$$

**Fig. 10:**
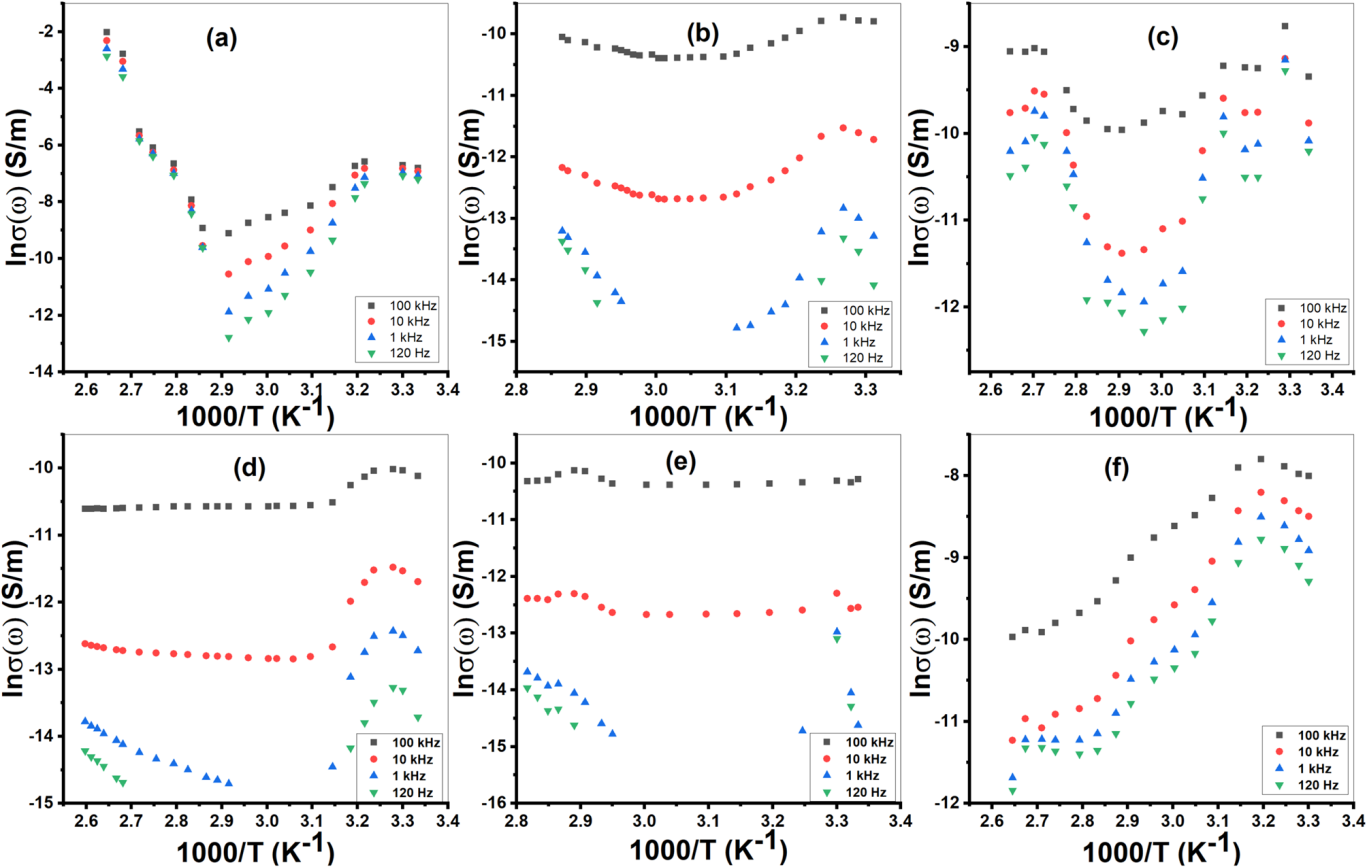
The plot of *lnσ (ω)*, against *1000/T (K*^−*1*^*)* for; (**a**) Et_2_DTC; (**b**) [Se(Et_2_DTC)_2_]; (**c**) [Ag(Et_2_DTC)]; (**d**) [CuSeO_3_H(Et_2_DTC)]; (**e**) [MnSeO_3_H(Et_2_DTC)]⋅H_2_O; (**f**) [Ag_2_Se(Et_2_DTC)_3_]⋅NO_3_.

Figure 10 exhibits the plot of *lnσ (ω)*, against *1000/T* for the investigated compounds. The overall trend of *lnσ (ω)* with *1000/T* shows an initial decrease, followed by an increase, then a gradual decrease again. At high *T*, the drop in *lnσ (ω)* value is due to band-transition.

The variation at the intermediate *T* range is probably due to the charge-carrier phonon scattering process. The decrease in the lower temperature range is considered to be due to an activation process. The value of *E* was calculated at high and low temperature ranges. The higher temperature *E* value is likely associated with a band to band transition process, whereas the lower temperature value is assumed to be due to an activation conduction process. Table 2 lists the computed activation energy values for 120 Hz, 1 kHz, 10 kHz, and 100 kHz. The *E* values vary at each range of temperature and at a certain frequency. This confirms the presence of more than one conduction mechanism as the temperature increases^41,42^.

**Table 2:** The activation energies E (eV) for samples at 120 Hz, 1 k Hz, 10 kHz and 100 kHz.

*F*	Et_2_DTC		[Se(Et_2_DTC)_2_]		[Ag(Et_2_DTC)]	[Ag_2_Se(Et_2_DTC)_3_].NO_3_	[CuSeO_3_H(Et_2_DTC)]		[MnSeO_3_H(Et_2_DTC)].H_2_O	
	*E*_*I*_	*E*_*II*_	*E*_*I*_	*E*_*II*_	*E*_*I*_	*E*_*I*_	*E*_*I*_	*E*_*II*_	*E*_*I*_	*E*_*II*_
100 kHz	0.153	2.713	0.017	0.218	0.270	0.176	0.170	–	–	0.187
10 kHz	0.064	2.697	0.061	0.336	1.159	0.242	0.344	0.035	0.701	0.358
1 kHz	–	2.629	0.075	1.201	1.365	0.329	0.479	0.247	4.283	0.937
120 Hz	–	2.544	0.051	1.713	1.277	0.408	0.730	0.491	4.667	0.991

**Fig. 11:**
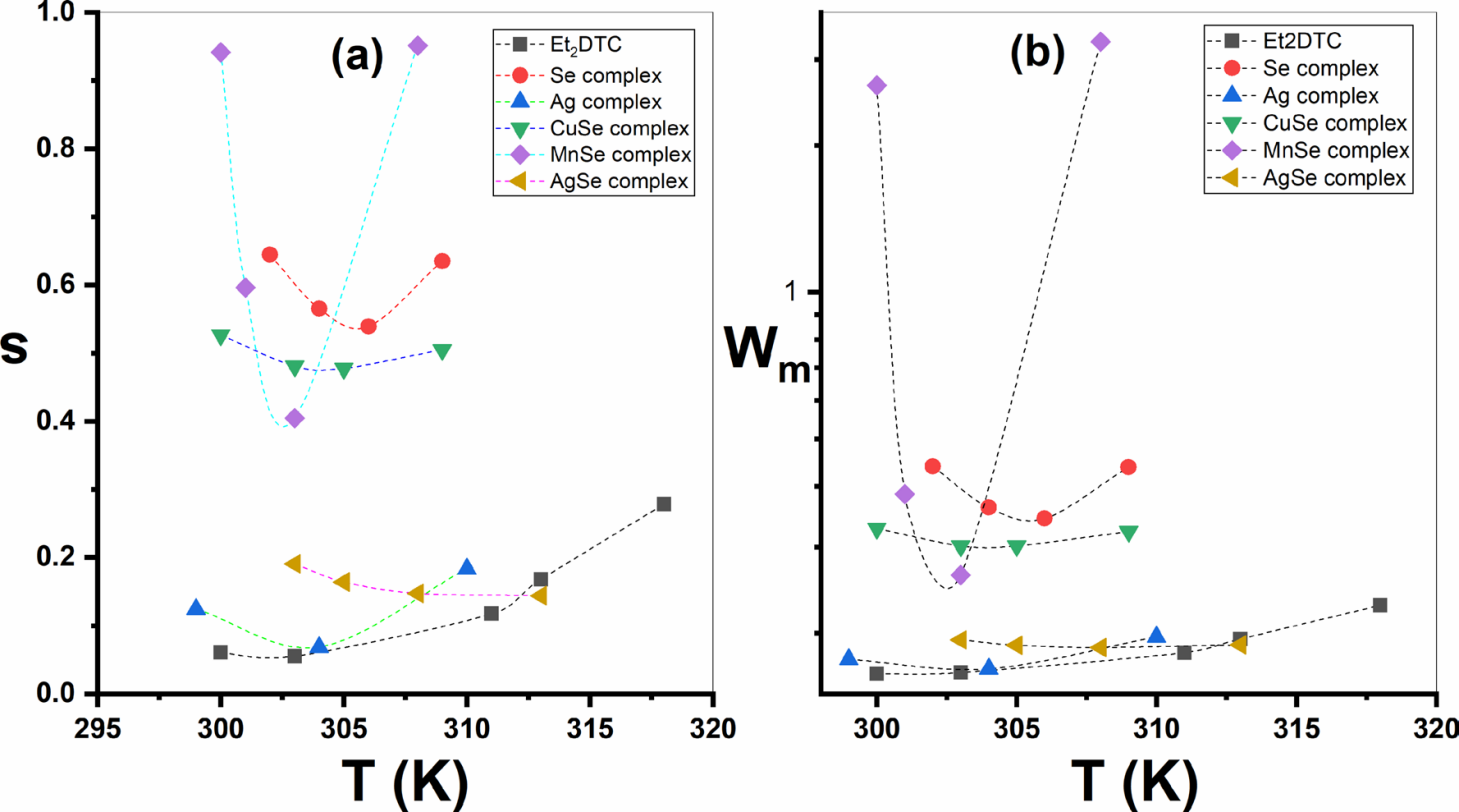
The variation of; (**a**) *s*, with *T* (K); (**b**) *W*_*m*_*, *with *T* (K) for samples.

Although the AC conductivity was measured at just a narrow frequency range (10^1^–10^5^ Hz), Fig. S6 presents a simplified plot of *ln σ (ω)* versus *ln (ω)*. The conduction process was examined using the following well-known relationship:

16 $$\sigma \left(\omega \right)=A{\omega }^{s}$$

where *A* represents a constant depending on *T* and *ω* represents the angular frequency. Further, *s* provides the exponent of frequency, which is less than or equal to one, indicating a dominant hopping process at such temperatures^43,44^. When *ln σ (ω)* and *ln (ω*) relation is plotted, the slope of the linear fit corresponds to the value of *s.* Figure 11a shows the variation of* s* with *T* for all samples. All samples exhibit a decrease in the exponent (*s*) with rising temperature at lower temperatures (300–318 K), followed by an increase at higher temperatures. So, this behavior can be explained by two-conduction mechanisms such as a CBH (Correlated-Barrier Hopping) and SPT (Small-Polaron Tunnelling)^45,46^. For investigated compounds, the maximum height of the barrier (*W*_*m*_) was determined utilizing the formula of CBH as follows^47^:

17 $$s=1-6{k}_{B}T/{W}_{m}$$

Figure 11b shows the relation between the values of *W*_*m*_ and T. All samples show a decrease in *W*_*m*_ value as the temperature rises, followed by an increase at higher *T*. It is observed that Et_2_DTC shows the lowest *W*_*m*_ value, whereas the [MnSeO_3_H(Et_2_DTC)].H_2_O complex displays the highest value of *W*_*m*_.

#### Dielectric properties

For the diethyldithiocarbamate and its metal-based complexes, the real part of the dielectric constant (*ε*_*r*_), dielectric loss (*ε*″), loss tangent (*tan δ*), and electric modulus (*M*) were determined as functions of both temperature and frequency. The experimental sample capacitance (*C*) is utilized to estimate the relative dielectric constant (real part of the dielectric) as follows^48^:

18 $${\varepsilon}_{r }=C d/{\varepsilon}_{0} A$$

where *d* and *A* are the sample dimensions, and *ε*_*0*_ is the permittivity of vacuum. At the frequencies under study, the imaginary component of the dielectric, dielectric loss (*ε*″), was estimated as follows^48^:

19 $${\varepsilon }^{{\prime}{\prime}}=G/\omega {C}_{0}$$

where the measured conductance is denoted by *G*. The expression for the loss tangent (tan δ) is as follows:

20 $$tan \delta = \varepsilon {\prime}{\prime}/{\varepsilon}_{r}$$

**Fig. 12:**
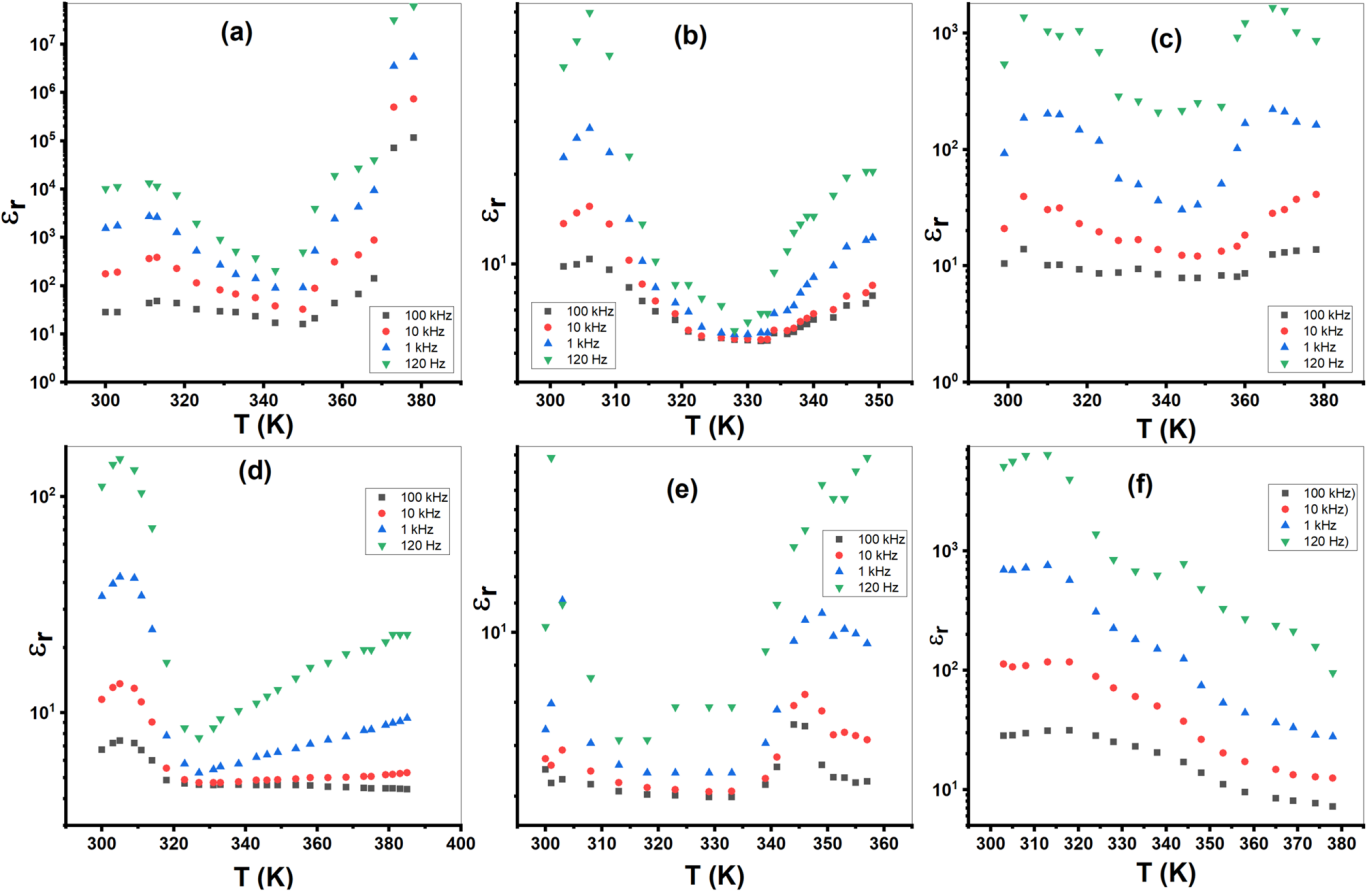
The plot of *ε*_*r*_ against *T* at the various frequencies; (**a**) Et_2_DTC; (**b**) [Se(Et_2_DTC)_2_]; (**c**) [Ag(Et_2_DTC)]; (**d**) [CuSeO_3_H(Et_2_DTC)]; (**e**) [MnSeO_3_H(Et_2_DTC)]⋅H_2_O; (**f**) [Ag_2_Se(Et_2_DTC)_3_]⋅NO_3_.

The complex electric modulus (M*) is a parameter used for evaluating the dielectric characteristics and AC conduction behavior of materials. The real component (*M*′) and imaginary component (*M*″) of electric modulus were determined from *ε*_*r*_ and *ε*′ data^48,49^. Figure 12 and Fig. S7 show the variation of *ε*_*r*_ and *ε*′ against *T* for the ligand (Et_2_DTC) and its investigated metal complexes. Both *ε*_*r*_ and *ε*′ increase with rising temperature, reaching their highest point at about 310 K for all samples. The initial rise in *ε*_*r*_ and *ε*′ with temperature is most likely caused by a hopping process and an increase in the amount of polarized charge carriers^12,50^. Consequently, all compounds show a decline in *ε*_*r*_ and *ε*′ at a specific temperature range, which can be the result of a decrease in polarized charges. This exhibits that these complexes undergo phase transition and undergo structural changes upon heating. One possible explanation for the increase of dielectric properties at high temperatures is associated with the emergence of a new phase transition, leading to a modification in the polarization mechanism^51,52^. Fig. S8 shows the peak behavior of *tan δ* with *T* at each frequency.

**Fig. 13:**
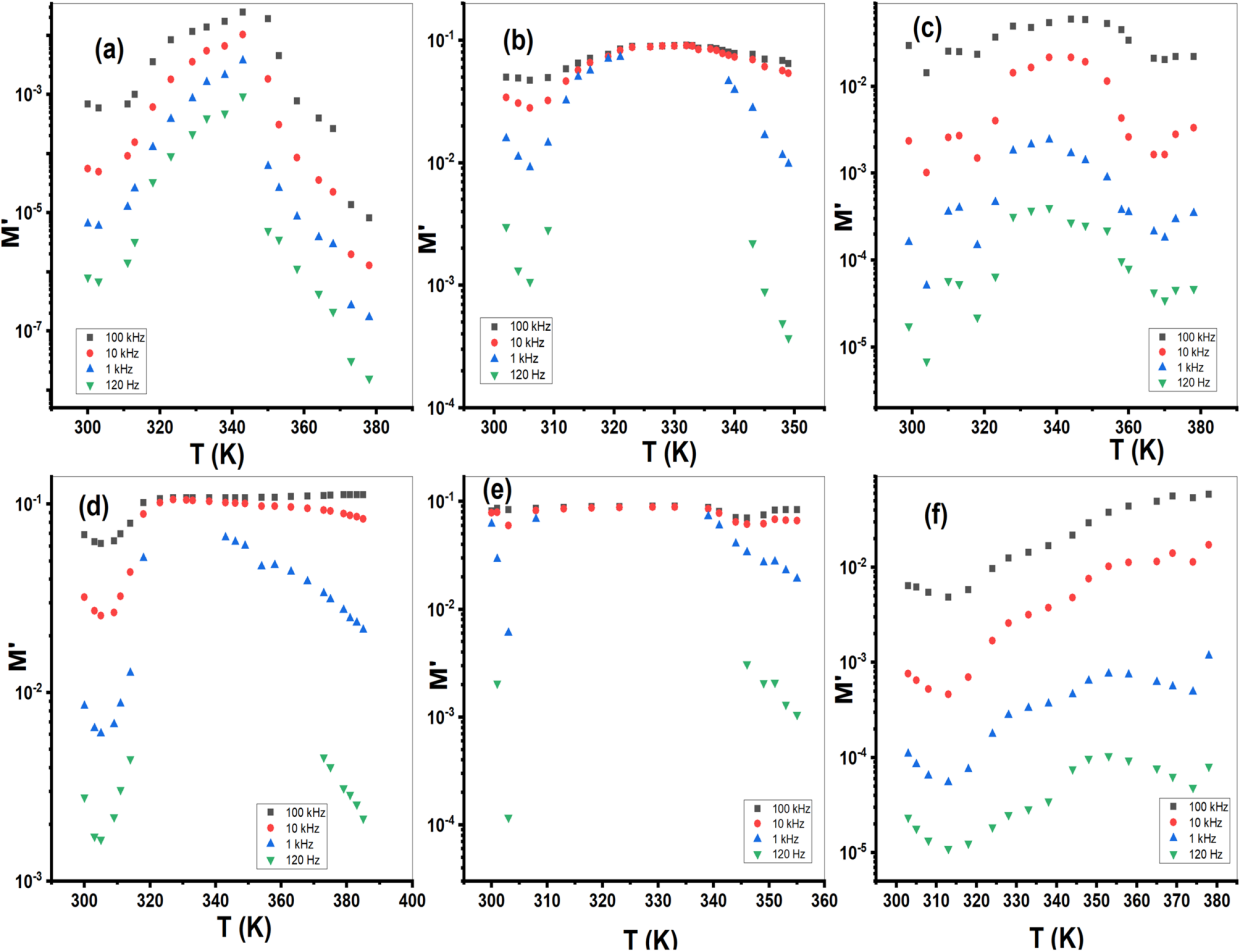
The plot of *M*′ versus temperature investigated frequencies for; (**a**) Et_2_DTC; (**b**) [Se(Et_2_DTC)_2_]; (**c**) [Ag(Et_2_DTC)]; (**d**) [CuSeO_3_H(Et_2_DTC)]; (**e**) [MnSeO_3_H(Et_2_DTC)]⋅H_2_O; (**f**) [Ag_2_Se(Et_2_DTC)_3_]⋅NO_3_.

Also, Fig. 13 and Fig. S9 show a peak behavior of (M′) and (M″) versus temperature at different frequencies. At low frequencies, the values of *M*′ and *M*″ tend to approach zero, indicating a decrease in electrical conductance. This behavior is consistent with the electrical conductivity measurements and suggests the presence of different conduction mechanisms^48^.

**Fig. 14:**
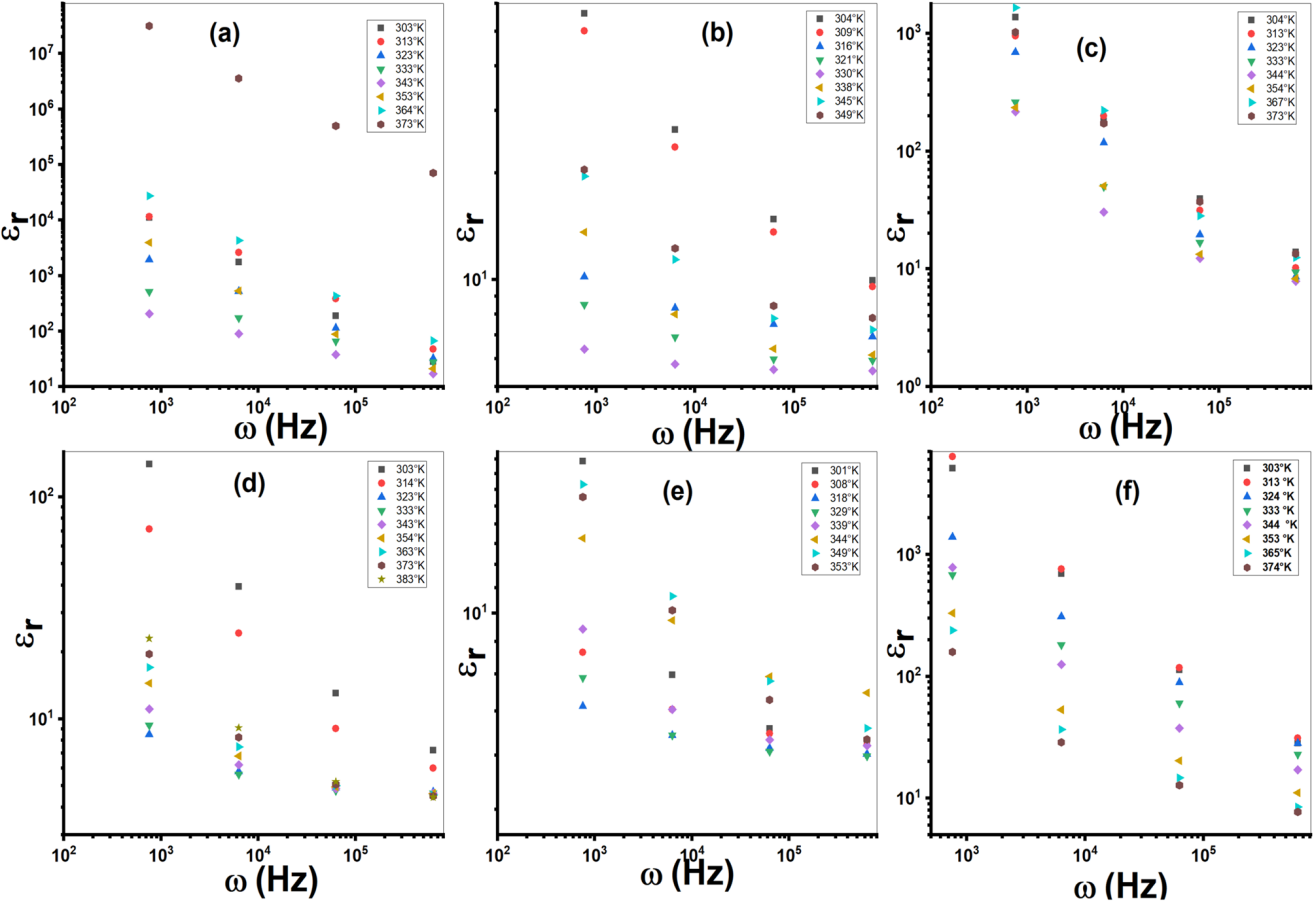
The plot of *ε*_*r*_ against *ω*; (**a**) Et_2_DTC; (**b**) [Se(Et_2_DTC)_2_]; (**c**) [Ag(Et_2_DTC)]; (**d**) [CuSeO_3_H(Et_2_DTC)]; (**e**) [MnSeO_3_H(Et_2_DTC)]**.**H_2_O; (**f**) [Ag_2_Se(Et_2_DTC)_3_]**.**NO_3_.

At an AC field, the* ε*_*r*_ represents the capacity of a substance for storing electrical energy, whereas the *tan δ* results from the passage of charged particles across the substance^53^. Figure 14 and Fig. S10 show the plot of *ε*_*r*_ and *tan δ* as function of *ω*, respectively. It is evident that the *ε*_*r*_ decreases with increasing frequency because the dipoles in polarizable substances lack the capacity to spin rapidly, causing their vibrations to lag after the fluctuating electric field^54^. While at low frequencies, the greater dielectric constant is caused by numerous polarization mechanisms, including electrode, interfacial, and orientational polarization^55^.

**Fig. 15:**
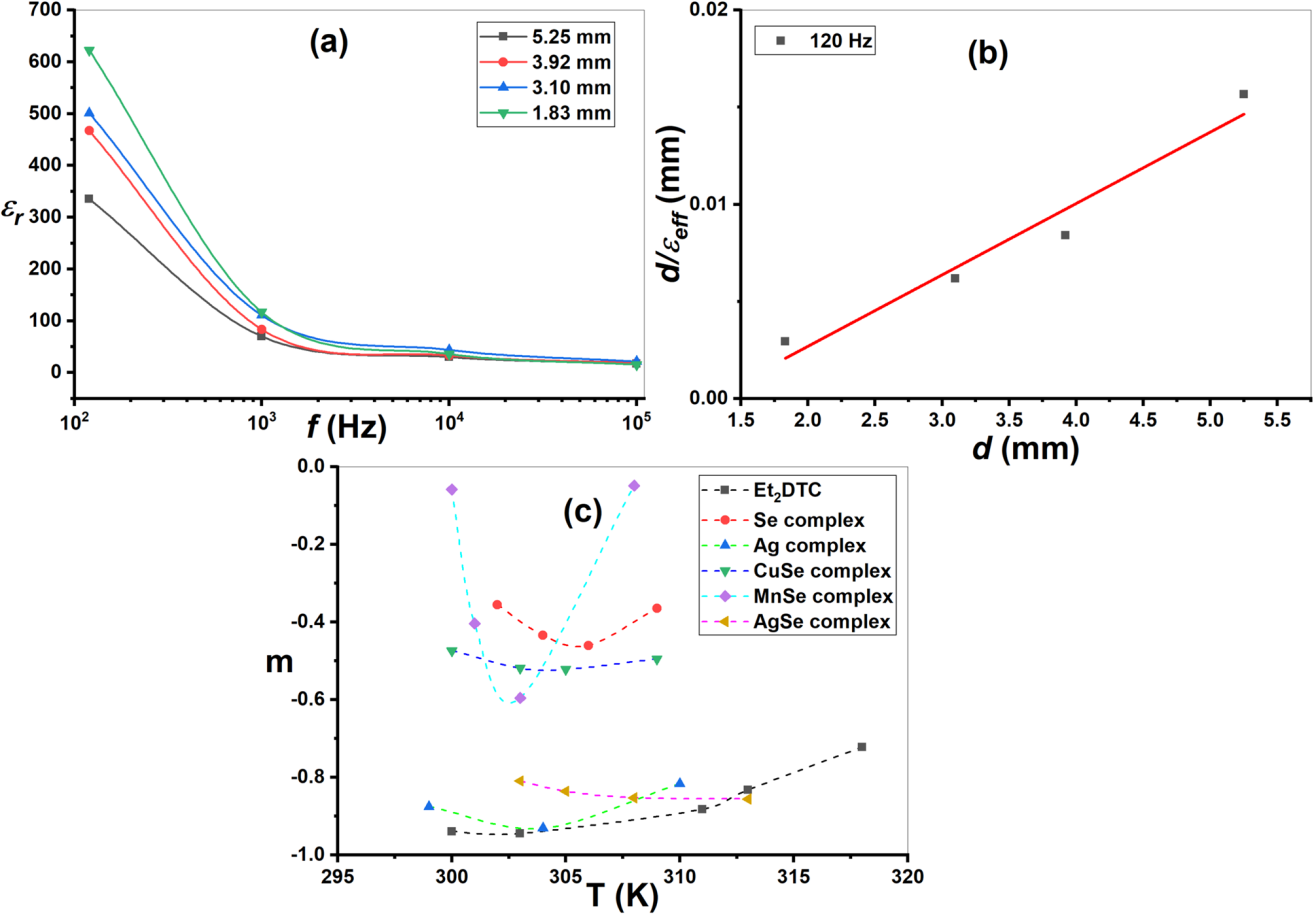
(**a**) Variation of *ε*_*r*_ with frequency *f* (Hz) at different specimen thickness of Et_2_DTC; (**b**) The d/ε_eff_ as a function of Et_2_DTC thickness d; (**c**) The variation of exponent (*m*) with temperature for Et_2_DTC ligand and its metal complexes.

In Fig. 15a, *ε*_*r*_ is plotted against the frequency *f,* for some selected thicknesses (*d*) of the Et_2_DTC sample. It is obvious that *ε*_*r*_ depends somehow on *d,* particularly at low frequencies^56^. The observed increase of the dielectric constant when the sample thickness decreases is not indeed a true intrinsic change, but rather an effect related to interfaces, electrode, and space-charge polarization. In interfacial (Maxwell–Wagner-Sillars) polarization, the electrode-sample interfaces play a much stronger role. In this case, charge can accumulate at grain boundaries, defects, and electrode-dielectric interfaces. This is well known as Maxwell–Wagner-Sillars (MWS) polarization^55,56^. Here, the effective dielectric constant, *ε*_*eff*_, is a summation of the bulk and the interface dielectric contributions; *ε*_*eff*_ = *ε*_*b*_ + *ε*_*i*_. The interface contribution becomes comparable to (or even may exceed) the bulk contribution, so the measured dielectric constant increases. As the sample becomes thinner, the relative contribution of the interfacial capacitance increases. The system now can be visualized as a combination of a series capacitances, bulk capacitor *C*_*b*_ and two interfacial capacitances, *C*_*i*_^57^:

21 $$\frac{1}{{C}_{eff}}=\frac{1}{{C}_{b}}+\frac{2}{{C}_{i}}$$

22 $${C}_{b}=\frac{{\varepsilon}_{b} A}{d},{C}_{i}=\frac{{\varepsilon}_{i} A}{{t}_{i}} \mathrm{a}\mathrm{n}\mathrm{d} {C}_{eff}=\frac{{\varepsilon}_{eff A}}{d}$$

where *A* and *d,* are the area and thickness of the sample, respectively and *t*_*i*_ is the interface thickness (which is practically very small). When the thickness of the sample, *d,* decreases, *C*_*b*_ increases. In this case, the interface capacitance *C*_*i*_ becomes dominant, and the total capacitance becomes more influenced by *C*_*i*_. The extracted dielectric constant appears larger. In addition, space-charge and electrode polarization may lead to enhancement in the measured dielectric constant. However, these are common in thin films^57^.

Using Eq. (22) in Eq. (21), a simplified form can be obtained as follows^57^:

23 $$\frac{d}{ {\varepsilon}_{eff}}=\frac{d}{{ \varepsilon }_{b}}+\frac{2{t}_{i}}{{ \varepsilon }_{i}}$$

To obtain a proper value of the bulk dielectric constant, *ε*_*b,*_ Eq. (23) was used and to plot $\frac{d}{ {\varepsilon}_{eff}}$ vs.* d* at low frequencies (120 Hz). The slope of the resulting straight-line yields *ε*_*b*_ which was found to be 271.74. In doped (impure) semiconductor systems, the high experimental values of the dielectric constant may be reduced by applying sufficient external magnetic field, depending on the impurity concentration^58^.

The simplified Giuntini relationship was applied at a certain temperature as follows^59^:

24 $${\varepsilon }^{{\prime}{\prime}}=B{\omega }^{m}$$

where *B* is a constant, and *m* is a frequency exponent associated with the transporting capabilities of substances^59^. Fig. S11 shows a simplified plot of *ln ε*″ versus *ln (ω)* and *m* is obtained from the slope. Figure 15 shows the plot of *m* versus *T* for diethyldithiocarbamate and its metal complexes. The suggested conduction processes are supported by the comparable performance of the *m* and *s* exponents.

### Thermal analysis

**Fig. 16:**
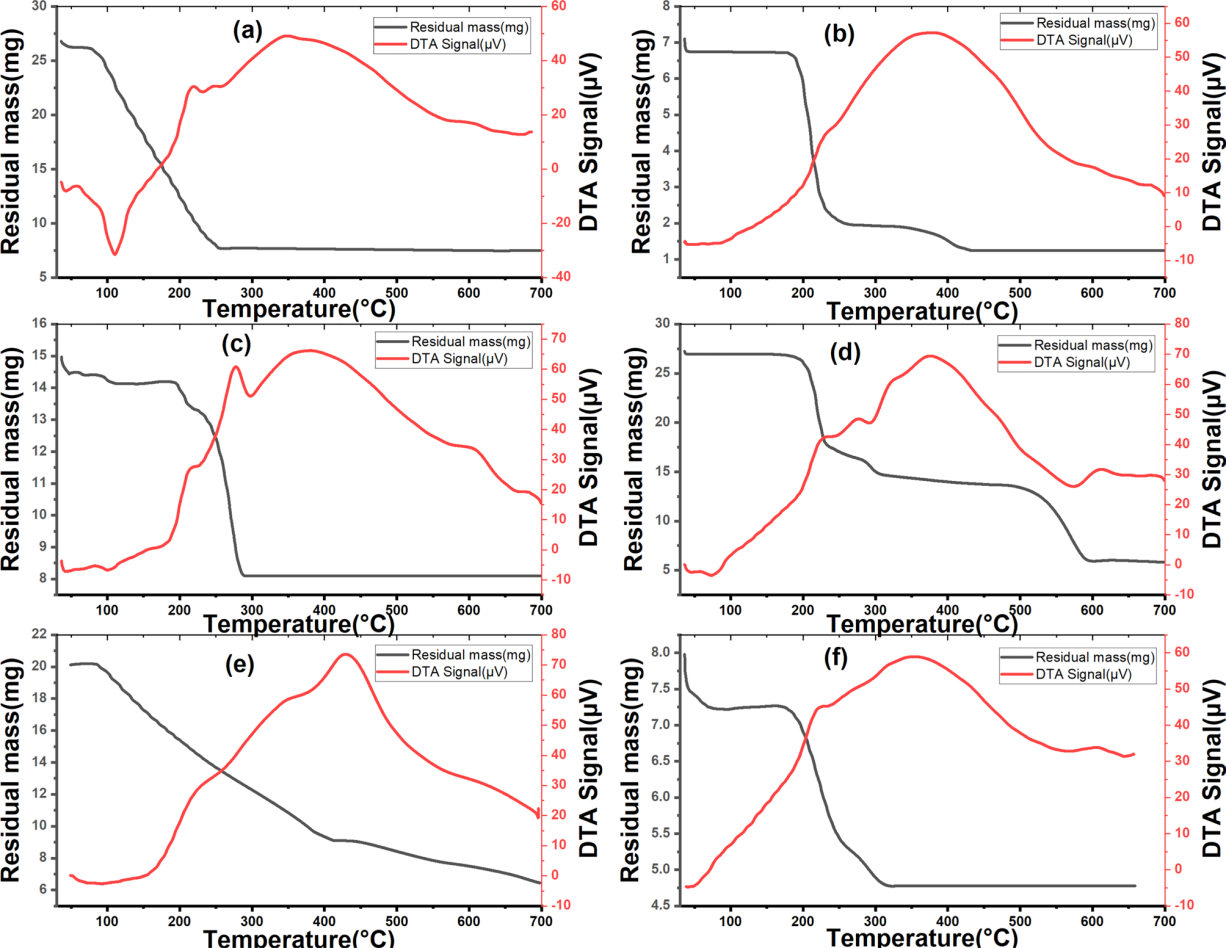
TGA and DTA for; (**a**) Et_2_DTC; (**b**) [Se(Et_2_DTC)_2_]; (**c**) [Ag(Et_2_DTC)]; (**d**) [MnSeO_3_H(Et_2_DTC)]⋅H_2_O; (**e**) [CuSeO_3_H(Et_2_DTC)]; (**f**) [Ag_2_Se(Et_2_DTC)_3_]⋅NO_3_.

Figure 16 shows the TGA and DTA of all studied compounds. It is observed that diethyldithiocarbamate (NaEt_2_DTC.3H_2_O) begin to decompose at around 100 ^ο^C due to the loss of water of hydration (Fig. 16a)^23^. After the dehydration process, diethyldithiocarbamate (Et₂DTC) decomposes continuously into volatile organic fragments such as CS_2_ and H_2_S within the temperature range of approximately 150–400 ⁰C, resulting in a mass loss of 48.31% and leaving a residue of Na_2_S. Similarly, the TGA curve of the mixed complex, [Ag_2_Se(Et_2_DTC)_3_].NO_3_, pointed to that first stage of weight loss (9.41%) takes place within the range 38.6–224.3 ^ο^C at peak temperature (*T*_m_ = 179.1 ^ο^C), Fig. 16f, corresponding to nitrate decomposition from the outer sphere as nitrogen oxides (Calc. 7.74%). The minor difference between the experimental and calculated weight losses could be attributed to a possible decomposition step occurring within the same temperature range as the onset of ligand (Et₂DTC) degradation. The [Ag(Et_2_DTC)] complex) exhibits a two-step decomposition pattern (Fig. 16c). In the first step, decomposition begins at a temperature range below 100 ⁰C, showing a small mass loss of 4.38% which corresponds to the removal of adsorbed water molecules. This behavior indicates the hygroscopic nature of this complex^60^. The second strong exothermic peak at *T*_m_ = 406.7 ^ο^C is accompanied by weight loss of 42.7% leaving a residue of Ag_2_S. Similarly, the [Se(Et_2_DTC)_2_] complex shows the same hygroscopic property, with mass loss of 3.79% assigned to adsorbed water removal. Nevertheless, the [Se(Et_2_DTC)_2_] complex undergoes two main successive decomposition steps within the temperature range of 179–700 ⁰C (Fig. 16b). In the first step (179–260 ⁰C), a weight loss of 70.7% corresponds primarily to the decomposition of approximately two ligand molecules (2 Et_2_NCS_2_^−^). In the final step (260–700 ⁰C), an additional weight loss of 11.0% is attributed to the elimination of the remaining ligand fragments and the partial volatilization of selenium, leaving a residue of about 17.9% as Se^0^ under an inert atmosphere (N_2_). In the case of [CuSeO_3_H(Et_2_DTC)] complex, a two-step decomposition mechanism is also observed (Fig. 16e). In the first step (48.2–408.3 ⁰C), a weight loss of 54.4% corresponds to the decomposition of the ligand (Et_2_NCS_2_^−^) and the partial evaporation of selenium. In the final step (408.3–697.6 ⁰C), the residual weight of 31.8% is consistent with the existence of CuS contaminated with CuSe. In contrast, the [MnSeO_3_H(Et_2_DTC)].H_2_O complex goes through a three-step decomposition process (Fig. 16d). In first step, the complex begins to lose weight at about 158.5 ⁰C-278.9 ⁰C due to dehydration of one water molecule and the partial decomposition of the diethyldithiocarbamate moiety, resulting in a weight loss 39.7%. The delayed removal of the lattice water may be attributed to the presence of an associated framework of complex molecules through inter- or intramolecular hydrogen bonding^60^. This step was followed by the loss of remaining ligand skeleton and conversion of selenium into SeO_2_ or volatile Se in the range 279.9–508.0 ⁰C, with a mass loss of 11.2%. In the final step (508.0–698.8 ⁰C), the residual mass (21.5%) is consistent with the formation of MnS containing a small amount of metallic Mn. It is worth mentioning that the formation of these sulfide residues at such elevated temperatures supports the high thermal stability of the investigated complexes^9^. Therefore, the [Ag(Et_2_DTC)] and [Ag_2_Se(Et_2_DTC)_3_].NO_3_ complexes may serve as effective single-source precursors of metal sulfides^23^.

#### Thermodynamic studies

Figure 16 displays the DTA peak temperature of studied compounds. According to the DTA peak analysis, the activation energy (*E*_*a*_) of thermolysis was obtained as follows^61^:

25 $$In \Delta t = C- {E}_{a}/RT$$

where *Δt* represents a distance from the baseline (^ο^C). When *ln Δt* was plotted versus *1/T* (Fig. S12), the slope provided the value of *E*_*a*_^61^. For each maximum temperature *T*_*m*_, the following equations were employed to determine the collision number (*Z*), activation entropy (*ΔS*^*#*^), and enthalpy (*∆H*^*#*^)^62^:

26 $$Z = {\varphi (E}_{a}/R{T}_{m}) exp\left({E}_{a}/R{T}_{m}^{2}\right)=\left({k}_{B} {T}_{m} /h\right) exp({ \Delta S}^{\#}/R)$$

27 $${\Delta S}^{\#} = {\Delta H}^{\#}/{T}_{m}$$

These thermodynamic parameters are listed for each compound in Table 3. It is evident that the thermolysis of diethyldithiocarbamate and its complexes is thermodynamically controlled. A direct correlation between the activation energy (*E*_*a*_) and the collision parameter (*Z*) was observed. In the final stage of the breakdown of the diethyldithiocarbamate complexes, the metal sulfide is formed, with an exothermic peak and the highest *E*_*a*_ value^23^. According to the negative sign of *ΔS*^*#*^, the transition states are characterized by a less random and more ordered molecular structure. The thermal conduct of all complexes was comparable, with their *ΔS*^*#*^ quantities lying within a narrow range (− 0.136 to − 0.154 kJ K^−1^ mol^−1^).

**Table 3:** Thermodynamic parameters of diethyldithiocarbamate and its based complexes.

Compound	Type	*T*_*m*_ (K)	*E*_*a*_ (kJ mol^−1^)	*ΔH*^*#*^ (kJ mol^−1^)	*Z* (s^−1^)	*ΔS*^*#*^ (kJ K^−1^ mol^−1^)
NaEt_2_DTC⋅3H_2_O	Endo	384	32.91	− 55.08	1.76	− 0.143
	Exo	625	58.50	− 86.68	1.91	− 0.139
[Se(Et_2_DTC)_2_]	Endo	440	11.66	− 66.96	0.53	− 0.152
	Exo	661	44.52	− 93.13	1.37	− 0.141
[Ag(Et_2_DTC)]	Endo	453	14.02	− 68.24	0.63	− 0.151
	Exo	680	43.09	− 96.00	1.28	− 0.141
[MnSeO_3_H(Et_2_DTC)]⋅H_2_O	Endo	353	31.85	− 50.69	1.86	− 0.144
	Endo	836	70.10	− 114.70	1.70	− 0.137
[CuSeO_3_H(Et_2_DTC)]	Endo	433	23.70	− 63.35	1.11	− 0.146
	Exo	707	84.67	− 95.79	2.45	− 0.136
[Ag_2_Se(Et_2_DTC)_3_]⋅NO_3_	Endo	452	9.16	− 69.70	0.41	− 0.154
	Exo	637	48.96	− 89.25	1.56	− 0.140

#### Differential scanning calorimetry (DSC)

Figures S13–S18 show the DSC curves of ligand (Et_2_DTC) and the five synthesized metal complexes. The heat capacity (*C*_P_) was estimated by dividing the heat flow by the rate of heating. The relationship between *C*_p_ and T can be demonstrated as follows^63^:

28 $${C}_{p} = aT + b,{C}_{p}/T= \alpha {T}^{2}+\gamma$$

where *a* and *b* are the parameters of Debye model, and *α* and *γ* represent the electronic and lattice heat capacity coefficients, respectively. Figs. S13-S18 show the plot of *C*_p_ versus *T* and *C*_p_*/T* against *T*^*2*^ for the studied compounds. Table S5 lists the Debye model coefficients over different temperature ranges, as well as the enthalpies values for each thermal transition. These transitions are attributed to dehydration, reversible phase transition, crystallization, and melting processes that occur prior to the complete decomposition of the investigated compounds^64^. Also, the entropy (*ΔS*^*#*^) of each thermal transition was calculated using Eq. (27). It was observed that the sign of *ΔS*^*#*^ is negative at the low temperature region of the exothermic peak, confirming that the crystallization and phase transition processes proceed through an ordered transition state.

### Structural optimization study

**Fig. 17:**
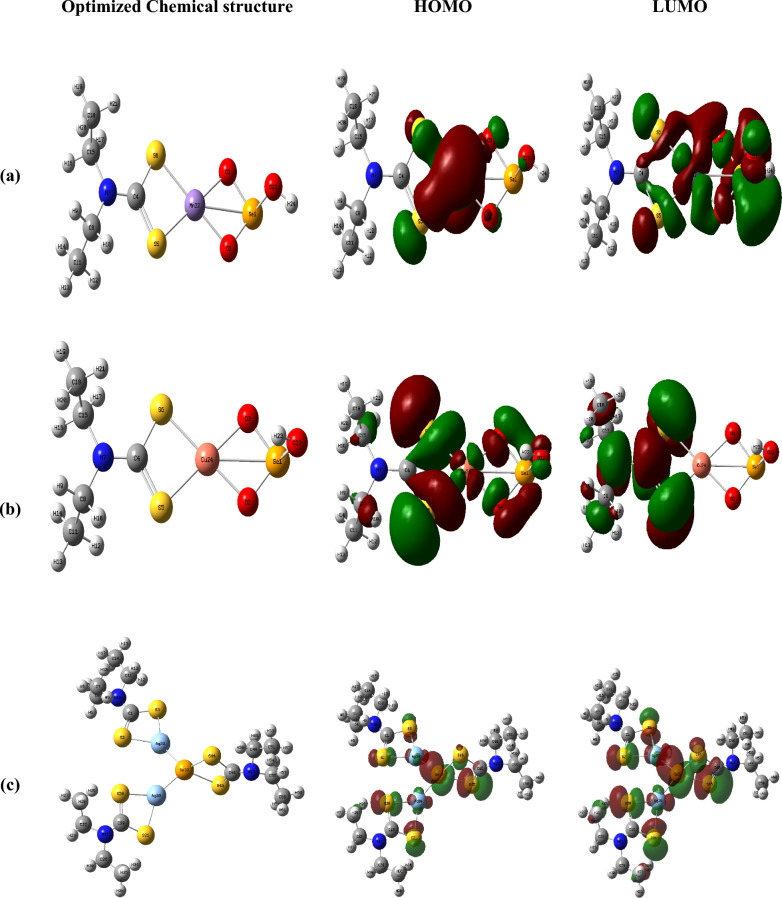
Optimized structures and contour plots frontier orbitals of mixed metal complexes; (**a**) [MnSeO_3_H(Et_2_DTC)]⋅H_2_O; (**b**) [CuSeO_3_H(Et_2_DTC)]; (**c**) [Ag_2_Se(Et_2_DTC)_3_]⋅NO_3_.

Density functional theory (DFT) calculations were performed to obtain the optimized molecular structures of the investigated complexes using DFT-B3LYP/ 6-31G basis set, implemented in the GAUSSIAN09 package and visualized by GAUSSVIEW^65,66^. The calculated theoretical parameters of the investigated complexes are summarized in Table S6, while the optimized structures and the HOMO–LUMO contour plots are presented in Fig. 17 and Fig. S19. The frontier molecular orbital distributions indicate that the HOMO and LUMO densities are mainly localized over the chelate region of the complexes. The optimized molecular structures of the mixed metal complexes [MnSeO_3_H(Et_2_DTC)].H_2_O and [CuSeO_3_H(Et_2_DTC)], (Fig. 17), display a bond between the selenium of biselenite and the metal ion (Cu or Mn) in addition to the bidentate bonding of the biselenite by the oxygen atoms, which is consistent with the FT-IR results as discussed above^16^. In addition, the optimized structure of the [Ag_2_Se(Et_2_DTC)_3_].NO_3_ complex indicates the presence of Ag–Se–Ag bridging bonding, resembling previously reported dithiocarbamate complexes such as [Pt_3_(S_2_CNEt_2_)_6_Ag_2_](ClO_4_)_2_, where a covalent bonding of two Ag (I) atoms to a square-planar Pt (II) was confirmed by single-crystal X-ray diffraction studies^67^. X-ray photoelectron spectroscopy (XPS) would provide direct confirmation of the selenium oxidation state in the Ag-Se complex. Table S6 summarizes the calculated HOMO–LUMO energy gaps (Δ*E*) of the investigated complexes. The HOMO–LUMO gap is widely regarded as an important parameter for estimating molecular softness and chemical reactivity, where smaller Δ*E* values generally indicate higher softness and enhanced electronic polarizability. Among the studied complexes, the Ag-Se complex exhibits the lowest energy gap (2.169 eV), suggesting greater electronic delocalization and higher chemical reactivity. This result is consistent with the electrical measurements, which show that Ag-Se complex possesses the lowest activation energy for electrical conduction, indicating facilitated charge transport within the system.

Additional molecular reactivity parameters have been estimated such as electronegativity (*χ*), chemical potential (*μ*), hardness (*η*), softness (*S*), and electrophilicity (*ω*), Table S6^64,68^. The highest softness value for Ag-Se complex (0.461 eV^−1^) indicates that this structure is more polarized and may demonstrate suitable nonlinear optical (NLO)^24^.

## Conclusion

The structural studies confirmed the ability of diethyldithiocarbamates to construct multinuclear assemblies with the investigated metal ions, resulting in sponge-like structure. The crystal structure of the samples was found to be polycrystalline with partial amorphous nature. FTIR analysis confirmed the bidentate bonding mode of diethyldithiocarbamate ligand through its two sulfur atoms. The UV–Visible spectra showed strong absorption in the range from 240 to 435 nm and high transmission (84%-99%) around 300 nm, with an optical band gap between 1.95 and 4.15 eV. Both the oscillator and dispersion energies of the linear refractive index (*n*) were obtained using the Wemple Di-Domenico single oscillator model. The emission spectra exhibited three fluorescence peaks in the range 427–531 nm. The electrical properties of diethyldithiocarbamate and its metal complexes showed semiconducting behavior with conductivity (*σ*_*ω*_) values ranging 10^–7^–10^–1^ *S/m*, and activation energies (*E*_*a*_) between 0.035–2.71 eV). Various conduction mechanisms were observed with increasing temperature, as confirmed by the changes of the dielectric parameters. TGA data emphasized that these complexes may serve as effective single-source precursors for the synthesis of metal sulfides on the nanoscale as semiconductor materials. Ultimately, the formation of these sulfide residues at such elevated temperatures supports the high thermal stability of the investigated complexes enhancing their potential for diverse technological and industrial uses such as optoelectronic devices, dielectric materials, catalysis, and sensing systems due to their tunable structural, optical, and electrical properties.

## Electronic Supplementary Material

Below is the link to the electronic supplementary material.

Supplementary Material 1

## Data Availability

The data supporting this article have been included as part of the Supplementary Information.
