# Supplementary material for: Structural, optical, electrical conductivity, and thermal properties of some mononuclear and mixed metal complexes of diethyldithiocarbamate
Source: Sci Rep. 2026 May 19;16:15465. doi: 10.1038/s41598-026-51751-0 (PMC13187047; doi:10.1038/s41598-026-51751-0)
Supplement: Supplementary file 1 — Supplementary Material 1 [file 41598_2026_51751_MOESM1_ESM.docx]

Supplementary data

**Structural, optical, electrical conductivity, and thermal properties of some mononuclear and mixed metal complexes of diethyldithiocarbamate**

**Rania Emara^1^*, Mamdouh S. Masoud^1^, Sayed Abboudy^2^ and Ahmed M. Ramadan^1^**

^1^Chemistry Department, Faculty of Science, Alexandria University, Alexandria, Egypt

^2^Physics Department, Faculty of Science, Alexandria University, Alexandria, Egypt

***Corresponding author,** Email address: [RaniaEmara@alexu.edu.eg](mailto:RaniaEmara@alexu.edu.eg)

**Table S1** Physical data of mononuclear complexes

| **Complex**  (M:L) | **Formula**  Color, m.p.°C | **% Calculated / (Found)** | | | | | **λ**  nm | ***μ*_eff_**  B.M | ***Λ*_m_**  Ω^-1^mol^-1^cm^2^ |
| --- | --- | --- | --- | --- | --- | --- | --- | --- | --- |
|  |  | **M** | **C** | **H** | **N** | **S** |  |  |  |
| **[Se(Et_2_DTC)_2_]**  (1:2) | **C_10_H_20_N_2_S_4_Se**  Yellow-orange, 85 | 21.03  (22.43) | 31.99  (32.14) | 5.37  (5.18) | 7.46  (7.65) | 34.16  (34.35) | 275,  335 | dia. | 1.89 |
| **[Ag(Et_2_DTC)]**  (1:1) | **C_5_H_10_NS_2_Ag**  yellow, 160 | 42.11  (42.36) | 23.45  (21.56) | 3.93  (3.98) | 5.47  (5.67) | 25.04  (23.39) | 274 | dia | 3.67 |

**Table S2** Physical data of mixed complexes

| **Complex**  (M:L) | **Formula**  Color, m.p.°C |  | **% Calculated / (Found)** | | | | | **λ**  nm | **μ_eff_**  B.M | ***Λ*_m_**  Ω^-1^mol^-1^cm^2^ |
| --- | --- | --- | --- | --- | --- | --- | --- | --- | --- | --- |
|  |  | **M_1_** | **M_2_** | **C** | **H** | **N** | **S** |  |  |  |
| **[MnSeO_3_H(Et_2_DTC)].H_2_O**  (1:1:1) | **C_5_H_13_NO_4_S_2_MnSe**  Off-white, 86 | 15.73  (15.95) | 22.61  (22.48) | 17.20 (17.28) | 3.75 (3.90) | 4.01 (4.29) | 18.37 (19.12) | 254, 279 | 7.23 | 3.82 |
| **[CuSeO_3_H(Et_2_DTC)]**  (1:1:1) | **C_5_H_11_NO_3_S_2_CuSe**  grey, 198 | 18.76 (25.13) | 23.31  (22.68) | 17.73 (17.41) | 4.68 (4.91) | 4.13 (4.08) | 18.93 (18.85) | 269, 433, | 2.52 | 2.33 |
| **[Ag_2_Se(Et_2_DTC)_3_].NO_3_**  (2:1:3) | **C_15_H_30_O_3_N_4_S_6_Ag_2_Se**  Buff, 160 | 26.92  (27.21) | 9.85  (10.10) | 22.48  (22.40) | 3.77  (4.93) | 6.99  (5.64) | 24.00  (24.38) | 275, 433 | Dia | 50.35 |

**Table S3** Fundamental IR absorption bands of the ligand and its complexes

| **Compound** | **ν (O–H)** | **ν (C-H)** | **ν (C–N)** | **ν (C–S)** | **ν_1_ (Se–O)** | **ν_2_ (Se–O)** |
| --- | --- | --- | --- | --- | --- | --- |
| **NaEt_2_DTC.3H_2_O** | 3367 | 2976 | 1480 | 988 | - | - |
| **Se(Et_2_DTC)_2_** | - | 2973 | 1491 | 973 | - | - |
| **Ag(Et_2_DTC)** | - | 2970 | 1489 | 973 | - | - |
| **[Ag_2_Se(Et_2_DTC)_3_].NO_3_** | - | 2970 | 1488 | 976 | - | - |
| **[MnSeO_3_H(Et_2_DTC)].H_2_O** | 3406 | 2974 | 1494 | 999 | 755 | 684 |
| **[CuSeO_3_H(Et_2_DTC)]** | 3501 | 2974 | 1561 | 993 | 776 | 708 |

**Table S4** XRD parameters of diethyldithiocarbamate metal complexes

| **Complex** | **Crystal system** | **Space group** | **Parameters of unit cell** | | | | | | ***Volume***  **(*Å^3^*)** | ***ε*** | **D (*nm*)** | |
| --- | --- | --- | --- | --- | --- | --- | --- | --- | --- | --- | --- | --- |
|  |  |  | ***a (Å)*** | ***b (Å)*** | ***c (Å)*** | ***α °*** | ***β °*** | ***ɣ °*** |  |  | **Scherrer** | **W-H** |
| **[Se(Et_2_DTC)_2_]** | Orthorhombic | P2_1_2_1_2_1_ (19) | 6.7 | 11.9 | 15.2 | 90 | 90 | 90 | 1229 | 0.002 | 30.76 | 59.25 |
| **[MnSeO_3_H(Et_2_DTC)].H_2_O** | Orthorhombic | Pmmm (47) | 19.0 | 9.0 | 5.9 | 90 | 90 | 90 | 1011 | 0.018 | 15.43 | 22.80 |
| **[CuSeO_3_H(Et_2_DTC)]** | Monoclinic | P12/m (10) | 11.2 | 10.3 | 6.9 | 90 | 91 | 90 | 797 | 0.005 | 17.77 | 25.21 |
| **[Ag(Et_2_DTC)]** | Monoclinic | P2_1_/c (14) | 10.9 | 24.7 | 11.5 | 90 | 126 | 90 | 2514 | 0.024 | 0.66 | 6.31 |
| **[Ag_2_Se(Et_2_DTC)_3_].NO_3_** | Cubic | Pm-3m (221) | 11.3 | 11.3 | 11.3 | 90 | 90 | 90 | 1439 | 0.094 | 1.19 | 1.46 |


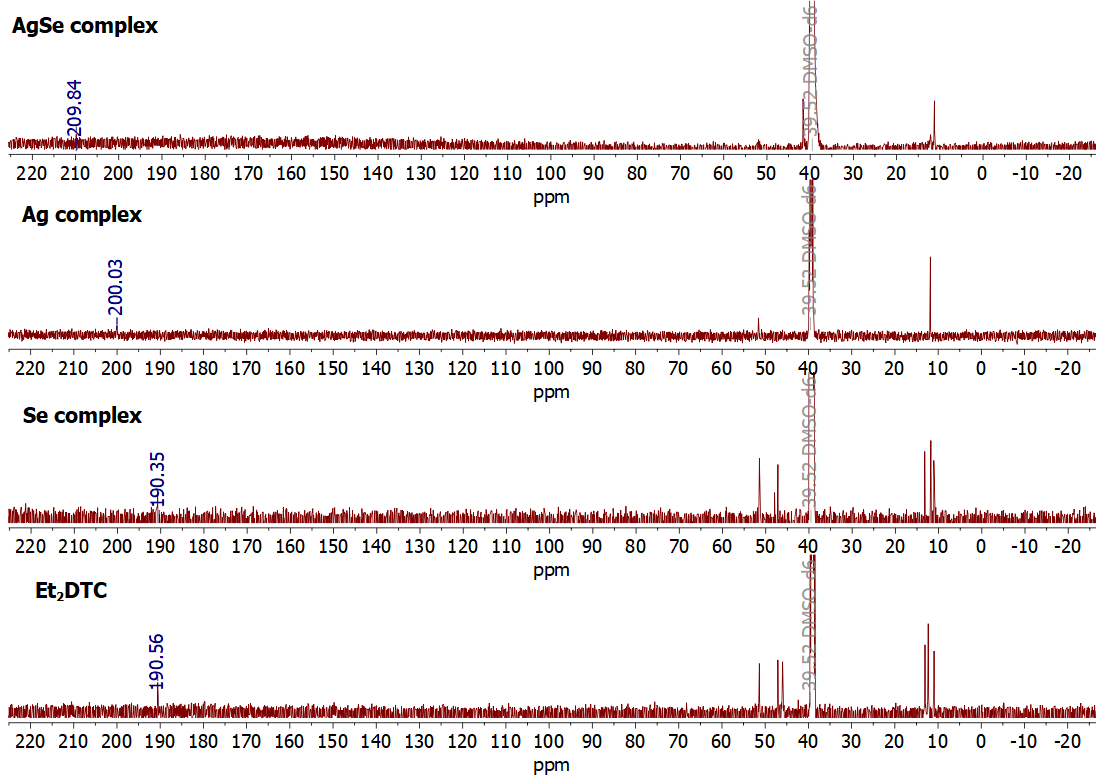


**Fig. S1** ^13^C NMR for ligand and diamagnetic complexes

**Fig. S2** Mass spectra of mixed complexes





**Fig. S3** The W–H curves of diethyldithiocarbamate complexes


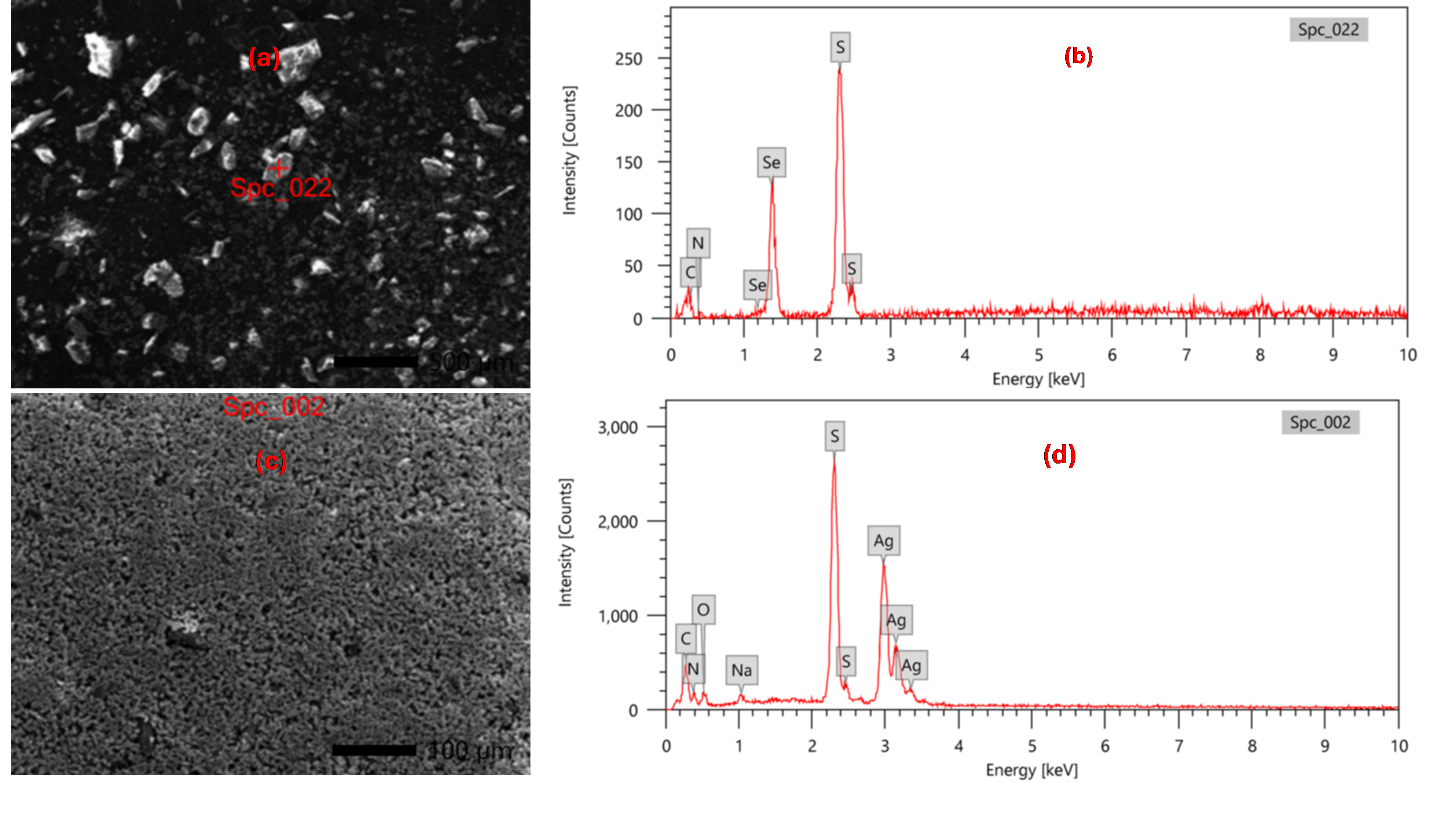


**Fig. S4** SEM and EDX analysis for; (a, b) [Se(Et_2_DTC)_2_]; (c, d) [Ag(Et_2_DTC)]





**Fig. S5** The variation of *σ (ω),* with *T (K)* at 120 Hz, 1 kHz, 10 kHz and 100 kHz for;(a) Et_2_DTC; (b) [Se(Et_2_DTC)_2_]; (c) [Ag(Et_2_DTC)]; (d) [CuSeO_3_H(Et_2_DTC)]; (e) [MnSeO_3_H(Et_2_DTC)].H_2_O; (f) [Ag_2_Se(Et_2_DTC)_3_].NO_3_





**Fig. S6** The variation of ln σ (ω), with ln ω (Hz) at the different temperatures for;(a) Et_2_DTC; (b) [Se(Et_2_DTC)_2_]; (c) [Ag(Et_2_DTC)]; (d) [CuSeO_3_H(Et_2_DTC)]; (e) [MnSeO_3_H(Et_2_DTC)].H_2_O; (f) [Ag_2_Se(Et_2_DTC)_3_].NO_3_





**Fig. S7** The variation of dielectric loss (ε'') with temperature at the different frequencies for; (a) Et_2_DTC; (b) [Se(Et_2_DTC)_2_]; (c) [Ag(Et_2_DTC)]; (d) [CuSeO_3_H(Et_2_DTC)]; (e) [MnSeO_3_H(Et_2_DTC)].H_2_O; (f) [Ag_2_Se(Et_2_DTC)_3_].NO_3_





**Fig. S8** The variation of Tan δ with temperature at the different frequencies for (a) Et_2_DTC; (b) [Se(Et_2_DTC)_2_]; (c) [Ag(Et_2_DTC)]; (d) [CuSeO_3_H(Et_2_DTC)]; (e) [MnSeO_3_H(Et_2_DTC)].H_2_O; (f) [Ag_2_Se(Et_2_DTC)_3_].NO_3_





**Fig. S9** The variation of imaginary electric modulus (M'') with temperature at different frequencies for; (a) Et_2_DTC; (b) [Se(Et_2_DTC)_2_]; (c) [Ag(Et_2_DTC)]; (d) [CuSeO_3_H(Et_2_DTC)]; (e) [MnSeO_3_H(Et_2_DTC)].H_2_O; (f) [Ag_2_Se(Et_2_DTC)_3_].NO_3_





**Fig. S10** The variation of Tanδ with temperature at different frequencies for; (a) Et_2_DTC; (b) [Se(Et_2_DTC)_2_]; (c) [Ag(Et_2_DTC)]; (d) [CuSeO_3_H(Et_2_DTC)]; (e) [MnSeO_3_H(Et_2_DTC)].H_2_O; (f) [Ag_2_Se(Et_2_DTC)_3_].NO_3_





**Fig. S11** The variation of ln ε'' with ln ω at the different temperatures for; (a) Et_2_DTC; (b) [Se(Et_2_DTC)_2_]; (c) [Ag(Et_2_DTC)]; (d) [CuSeO_3_H(Et_2_DTC)]; (e) [MnSeO_3_H(Et_2_DTC)].H_2_O; (f) [Ag_2_Se(Et_2_DTC)_3_].NO_3_





**Fig. S12** DTA analysis of diethyldithiocarbamate (a) and its complexes (b-f)





**Fig. S13** DSC and Debye model for Et_2_DTC





**Fig. S14** DSC and Debye model for Se complex





**Fig. S15** DSC and Debye model for Ag complex





**Fig. S16** DSC and Debye model for MnSe complex





**Fig. S17** DSC and Debye model for CuSe complex





**Fig. S18** DSC and Debye model for AgSe complex

**Table S5** DSC thermal transition and Debye models parameters of diethyldithiocarbamate and some of its complexes

| **Compound** | **Temperature range (◦C)** | **T_m_**  **(⁰C)** | **ΔH**  **J/g** | **ΔS**  **J/g ⁰C** | **C_p_ = a T + b** | | | **C_p_/T = α T + γ** | | |
| --- | --- | --- | --- | --- | --- | --- | --- | --- | --- | --- |
|  |  |  |  |  | **a** | **b** | **R^2^** | **α x10^-6^** | **ɣ** | **R^2^** |
| **Et_2_DTC** | 24-112  112-485  485-690 | 110.7  261.0  686.6 | -703.01  -101.68  -13.59 | -6.35  -0.39  -0.02 | -1.87  2.49  0.30 | 562.03  -1108.55  -52.42 | 0.834  0.973  0.966 | -7.28  6.47  0.07 | 0.64  -1.29  0.19 | 0.859  0.962  0.480 |
| **[Se(Et_2_DTC)_2_]** | 18-70  70-188  188-462  462-700 | -  90.5  350.9  535.1 | -  -1331.44  561.30  -386.37 | -  -14.71  1.59  -0.72 | -0.61  0.69  0.68  0.03 | 168.04  -283.44  -242.31  201.45 | 0.856  0.974  0.947  0.063 | -2.57  1.91  0.50  -0.16 | 0.18  -0.32  0.09  0.38 | 0.762  0.937  0.775  0.784 |
| **[Ag(Et_2_DTC)]** | 25-112  112-195  195-278  278-305  305-615  615-700 | -  184.9  -  -  461.6  - | -  -1690.75  -  -  1355.78  - | -  -0.87  -  -  2.94  - | -0.155  0.55  2.46  -1.02  0.32  -0.50 | 14.45  -246.16  -1120.84  783.95  49.55  751.28 | 0.315  0.978  0.900  0.356  0.901  0.724 | -2.35  2.14  4.18  -4.11  -0.05  -0.44 | 0.14  -0.41  -0.84  1.69  0.41  0.68 | 0.505  0.952  0.923  0.787  0.141  0.838 |
| **[MnSeO_3_H(Et_2_DTC)].H_2_O** | 34-82  82-230  230-313  313-379  379-583  583-700 | 77.6  -  296.2  -  566.1  - | -190.23  -  -103.93  -  -354.94  - | -2.45  -  -0.35  -  -0.63  - | -0.35  1.24  -0.06  0.93  -0.15  0.58 | 97.28  -450.26  212.95  -331.18  379.30  -213.76 | 0.788  0.992  0.018  0.966  0.688  0.905 | 0.52  2.54  -0.70  0.87  -0.46  0.16 | -0.10  -0.28  0.53  0.06  0.61  0.21 | 0.076  0.970  0.727  0.937  0.932  0.606 |
| **[CuSeO_3_H(Et_2_DTC)]** | 47-118  118-207  207-394  394-431  431-506  506-681 | -  181.4  -  -  499.8  - | -  -1435.07  -  -  -476.86  - | -  -7.91  -  -  -0.95  - | -0.25  1.06  0.92  2.73  -0.96  0.23 | 70.85  -441.44  -367.23  -1563.94  1040.74  117.52 | 0.577  0.818  0.994  0.690  0.951  0.932 | -0.66  2.76  0.89  4.80  -1.33  -0.09 | 0.03  -0.48  -0.02  -1.80  1.17  0.44 | 0.330  0.855  0.972  0.956  0.963  0.845 |
| **[Ag_2_Se(Et_2_DTC)_3_].NO_3_** | 19-60  60-231  231-479  479-658 | 58.9  -  465.5  - | -747.55  -  -1302.08  - | -12.69  -  -2.80  - | -0.43  1.30  0.13  0.62 | 121.54  -447.61  127.23  -227.57 | 0.535  0.992  0.896  0.972 | 1.70  3.09  -0.30  0.20 | -0.21  -0.32  0.45  0.19 | 0.293  0.973  0.916  0.876 |

**Table S6** The molecular parameters of the synthesized complexes

| **Compound** | ***E*_HOMO_ (eV)** | ***E*_LUMO_**  **(eV)** | **Δ*E***  **(eV)** | ***η***  **(eV)** | ***S***  **(eV^-1^)** | ***χ***  **(eV)** | ***μ***  **(eV)** | ***ω***  **(eV)** |
| --- | --- | --- | --- | --- | --- | --- | --- | --- |
| **Se complex** | -5.431 | -1.793 | 3.638 | 1.819 | 0.275 | 3.612 | -3.612 | 3.586 |
| **Ag complex** | -5.448 | -2.573 | 2.875 | 1.438 | 0.348 | 4.010 | -4.010 | 5.592 |
| **MnSe complex** | -5.485 | -1.518 | 3.967 | 1.980 | 0.252 | 3.502 | -3.502 | 3.096 |
| **CuSe complex** | -6.063 | -1.334 | 4.729 | 2.364 | 0.211 | 3.698 | -3.698 | 2.892 |
| **AgSe complex** | -5.154 | -2.985 | 2.169 | 1.084 | 0.461 | 4.070 | -4.070 | 7.639 |

**Fig. S19** Optimized structures and contour plots frontier orbitals of mononuclear metal complexes; (a) [Se(Et_2_DTC)_2_]; (b) [Ag(Et_2_DTC)]
